# Complete genome analysis of sugarcane root associated endophytic diazotroph *Pseudomonas aeruginosa* DJ06 revealing versatile molecular mechanism involved in sugarcane development

**DOI:** 10.3389/fmicb.2023.1096754

**Published:** 2023-04-20

**Authors:** Dao-Jun Guo, Pratiksha Singh, Bin Yang, Rajesh Kumar Singh, Krishan K. Verma, Anjney Sharma, Qaisar Khan, Ying Qin, Ting-Su Chen, Xiu-Peng Song, Bao-Qing Zhang, Dong-Ping Li, Yang-Rui Li

**Affiliations:** ^1^College of Life Sciences and Engineering, Hexi University, Zhangye, Gansu, China; ^2^Key Laboratory of Sugarcane Biotechnology and Genetic Improvement (Guangxi), Ministry of Agriculture, Guangxi Key Laboratory of Sugarcane Genetic Improvement, Sugarcane Research Institute, Guangxi Academy of Agricultural Sciences, Nanning, Guangxi, China; ^3^College of Agriculture, Guangxi University, Nanning, Guangxi, China; ^4^Microbiology Institute, Guangxi Academy of Agricultural Sciences, Nanning, Guangxi, China

**Keywords:** *Pseudomonas aeruginosa*, endophyte, complete genome, PGPB, root colonization, sugarcane

## Abstract

Sugarcane is an important sugar and bioenergy source and a significant component of the economy in various countries in arid and semiarid. It requires more synthetic fertilizers and fungicides during growth and development. However, the excess use of synthetic fertilizers and fungicides causes environmental pollution and affects cane quality and productivity. Plant growth-promoting bacteria (PGPB) indirectly or directly promote plant growth in various ways. In this study, 22 PGPB strains were isolated from the roots of the sugarcane variety GT42. After screening of plant growth-promoting (PGP) traits, it was found that the DJ06 strain had the most potent PGP activity, which was identified as *Pseudomonas aeruginosa* by 16S rRNA gene sequencing. Scanning electron microscopy (SEM) and green fluorescent protein (GFP) labeling technology confirmed that the DJ06 strain successfully colonized sugarcane tissues. The complete genome sequencing of the DJ06 strain was performed using Nanopore and Illumina sequencing platforms. The results showed that the DJ06 strain genome size was 64,90,034 bp with a G+C content of 66.34%, including 5,912 protein-coding genes (CDSs) and 12 *rRNA* genes. A series of genes related to plant growth promotion was observed, such as nitrogen fixation, ammonia assimilation, siderophore, 1-aminocyclopropane-1-carboxylic acid (ACC), deaminase, indole-3-acetic acid (IAA) production, auxin biosynthesis, phosphate metabolism, hydrolase, biocontrol, and tolerance to abiotic stresses. In addition, the effect of the DJ06 strain was also evaluated by inoculation in two sugarcane varieties GT11 and B8. The length of the plant was increased significantly by 32.43 and 12.66% and fresh weight by 89.87 and 135.71% in sugarcane GT11 and B8 at 60 days after inoculation. The photosynthetic leaf gas exchange also increased significantly compared with the control plants. The content of indole-3-acetic acid (IAA) was enhanced and gibberellins (GA) and abscisic acid (ABA) were reduced in response to inoculation of the DJ06 strain as compared with control in two sugarcane varieties. The enzymatic activities of oxidative, nitrogen metabolism, and hydrolases were also changed dramatically in both sugarcane varieties with inoculation of the DJ06 strain. These findings provide better insights into the interactive action mechanisms of the *P. aeruginosa* DJ06 strain and sugarcane plant development.

## Introduction

Sugarcane is an important C4 crop with great potential to contribute to biofuel production worldwide. It requires a large number of fertilizers, herbicides, and fungicides for plant growth and development (Li and Yang, 2015; Wayment et al., 2021). However, the application of chemical fertilizers could increase crop yields but has dramatically harmed the farmland environment and human health (Savci, 2012; Feng et al., 2020; Meftaul et al., 2020). Plant growth-promoting bacteria (PGPB) are the beneficial microorganisms that colonize plant tissues and symbiotically coexist with plants to promote plant growth through nitrogen fixation, secretion of siderophore, biocontrol, and improvement of plant resistance to stresses (Kloepper et al., 1980; Nehra and Choudhary, 2015; García et al., 2017; Singh et al., 2017; Fukami et al., 2018).

Biological nitrogen fixation (BNF) is the best way to reduce the application of chemical nitrogen fertilizer in crop production. The nitrogen contribution level in sugarcane reached 18–57.31%, verified by the ^15^N natural abundance technique after inoculating nitrogen-fixing bacteria, including *Herbaspirillum seropedicae* IPA-CC9, *Pseudomonas* sp. IPA-CC33, and *Bacillus megaterium* IPA-CF6 (Antunes et al., 2019). The nitrogen uptake of sugarcane varieties, GT11 and B8, has significantly improved on inoculation of *Enterobacter roggenkampii* ED5 strain, and physiological enzymatic activities were also considerably changed (Guo et al., 2021). Some PGPBs were used for biocontrol due to having an effective inhibitory effect on plant pathogens. In an earlier study, Singh et al. (2021) reported that *Pseudomonas aeruginosa* B18 enhanced the growth of the sugarcane variety Yacheng 71-374 in response to the smut pathogen *Sporisorium scitamineum*. Similarly, sugarcane pathogens *Colletotrichum falcatum* and *Fusarium moniliforme* were controlled by applying *B. amyloliquefacien*s and *B. gladioli* CP2 (Bharathalakshmi and Jamuna, 2019; Pitiwittayakul et al., 2021). Improving plant resistance against abiotic stresses is one of the main growth-promoting properties of PGPB. For example, the salt tolerance of sugarcane was enhanced with *B. xiamenensis* ASN-1 inoculation (Sharma et al., 2021). The nitrogen-fixing strain *Streptomyces chartreuses* WZS021 could effectively improve the drought resistance of sugarcane (Wang et al., 2019).

In recent years, some sugarcane rhizosphere or endogenous growth-promoting strains were isolated, such as *Kosakonia radicincitans, Stenotrophomonas maltophilia, Herbaspirillum seropedicae, Enterobacter roggenkampii, P. entomophila, Klebsiella variicola, Paenibacillus lactis, B. xiamenensis, Klebsiella pneumonia, Gluconacetobacter diazotrophicus*, etc. (Mirza et al., 2001; Wei et al., 2014; Lamizadeh et al., 2016; Bhardwaj et al., 2017; Li et al., 2017; Oliveira et al., 2018; Guo et al., 2020; Singh et al., 2020; Xia et al., 2020). Whereas, *Pseudomonas* is a common PGPB with broad application prospects, and the inoculation of *P. fluorescens* promoted phosphorus uptake in sugarcane, thereby promoting its proper growth (Rosa et al., 2022).

The whole genome sequencing technology provides a suitable method for revealing the molecular mechanism of interaction between PGPB and crops. In our laboratory, the complete genome sequence of the sugarcane endophytic nitrogen-fixing bacteria *E. roggenkampii* ED5 was analyzed, and a variety of potential genes related to auxin, siderophore, nitrogen metabolism, and resistance to abiotic stresses were found in the genome of the strain, revealing the molecular mechanisms of plant growth promoting effects of the strain on sugarcane (Guo et al., 2020). The potential biocontrol mechanism of *Streptomyces griseorubiginosus* BTU6 was shown by genome sequencing (Wang et al., 2021). Although research has been conducted on *Pseudomonas* rhizosphere strains in sugarcane, most of them are from soils, but studies on the endophytic *Pseudomonas* strains are still limited (Singh et al., 2021). Meanwhile, the molecular basis of *Pseudomonas* for promoting sugarcane growth remains unclear.

In the present study, whole-genome sequencing technology was applied to analyze the molecular basis of sugarcane growth promotion by endophytic *Pseudomonas* strain isolated from sugarcane root. The purpose of this study was (i) isolation of endophytic bacterial strains from sugarcane variety GT42 roots and analyze their plant growth-promoting (PGP) properties, (ii) observation of the colonization of the *P. aeruginosa* DJ06 strain in sugarcane tissue through scanning electron microscope (SEM) and confocal laser scanning microscope (CLSM), (iii) sequencing the complete genome of *P. aeruginosa* DJ06 and predict its potential PGP-related genes, (iv) investigation of the agronomic traits and photosynthetic responses of sugarcane varieties GT11 and B8 after inoculation of *P. aeruginosa* DJ06, and (v) the analysis of the changes in endogenous phytohormones and physiological enzymes of sugarcane responsive to *P. aeruginosa* DJ06 inoculation.

## Materials and methods

### Isolation of endophytic bacteria from sugarcane

Endophytic bacterial strains were isolated from the sugarcane variety GT42, which has the most extensive planting area in China at present. The sugarcane roots were rinsed with tap water; 1 g of root sample was cut into 5 mm segments and soaked in 4% sodium hypochlorite solution for 2 min and alcohol (75%) for 2 min; and then washed with sterile water three times and used the water as the control for final washing. The cleaned root segments were put into a sterilized mortar and ground into a powder with a pestle, adding 10 ml of sterile water to dissolve it completely and placing it for 30 min. The suspension was diluted (10^−1^, 10^−2^, 10^−3^, 10^−4^, 10^−5^, and 10^−6^), and 100 μl of each diluted suspension was drawn and spread evenly on five different media plates, respectively (Supplementary Table 1), and incubated at 32°C for 48 h. No colonies appeared in the control plates, which means the isolation of endophytic bacteria in the sugarcane roots was successful. The single colonies were picked for purification, and the purified strains were stored at −80°C with 20% glycerol for later use.

### DNA extraction from isolated strains and identification

High-quality genomic DNA of the strains was extracted with a QIAGEN QIAmp^®^ DNA extraction kit. The 16S rRNA gene was amplified, and high-quality amplified products were obtained by 1% agarose gel detection for purification and sequencing (Li et al., 2017). The PCR primers and reaction conditions are presented in Table 1. The Trelief^®^ DNA Gel Extraction Kit (Tsingke, China) was used for PCR product purification. The 16S rRNA gene sequences of all the endophytic strains were sequenced by Tsingke Biotechnology Co. Ltd., Beijing, China. The 16S rRNA gene was sequenced and spliced with ContigExpress software, BLAST-similar sequence alignment was performed in NCBI for each strain, and the specific sequence was submitted to the NCBI GenBank database to obtain the accession number.

**Table 1:** Primer sequences used for 16S rRNA gene amplification.

**Gene**	**Primer**	**Sequence (5^′^ → 3^′^)**	**Product size (bp)**	**PCR conditions**	**Reference**
*16S rRNA*	pA-F pH-R	AGAGTTTGATCCTGGCTCAG AAGGAGGTGATCCAGCCGCA	1300 to 1600	Initial temperature (95°C for 5 min), start cycles (30), denaturation (95°C for 1 min), annealing (55°C for 1 min), elongation (72°C for 1 min), final extension (72°C for 5 min).	Edwards et al. (1989)

### Screening for PGP activities

The PGP activities including phosphate (P) solubilization, hydrogen cyanide (HCN), siderophore, DF-ACC (1-aminocyclopropane-1-carboxylic acid), and ammonia production were examined. All the inoculated strains were fresh bacteria cultured in liquid for 3 days, and all the experiments were performed in three biological replicates.

#### Phosphate solubilization

Pikovskaya (1948) agar plate was used to test the activity of dissolved inorganic phosphorus. A total of 5 μl of each bacterial solution was pipetted and inoculated in the center of Pikovskaya's agar culture plates, and each culture plate was sealed with parafilm and placed in an incubator at 32°C for 72 h. The appearance of a colorless transparent circle indicates that the strain can dissolve phosphorus, and the strength of the ability to dissolve phosphorus is positively correlated with the diameter of the colorless transparent circle.

#### Hydrogen cyanide production

The strains were inoculated into the nutrient broth (NB) tubes with 4.4 g L^−1^ of glycine, and the filter paper strip (0.5 cm × 8 cm) was soaked in 1% picric acid solution for 10 min, and then hung on the culture tubes and sealed. It was incubated at 32°C in a dark incubator for 4–7 days. If the color of the filter paper changed, it confirmed that the strain could produce HCN. The color of the filter paper indicated the strength of the produced HCN (Lorck, 1948).

#### DF-ACC test

A total of 3.0 mmol of 1-aminocyclopropane-1-carboxylic acid (ACC) was added to 1 L DF medium (Jacobson et al., 1994), to make culture plates for qualitative analysis. The strains were inoculated on the DF-ACC plates and cultured at 32°C for 72 h. The ability of the strain to utilize an ACC nitrogen source was positively correlated with the diameter of the bacterial circle (Li et al., 2011).

#### Siderophore production

The ability of siderophore production by strains was tested by the chrome azurol S (CAS) plates method (Schwyn and Neilands, 1987). A total of 5 μl sample of each bacterial solution was tested, inoculated in the center of CAS plates, sealed with parafilm, and then placed in the incubator at 32°C for 72 h for observation. The siderophore production ability of strains was positively correlated with the diameter of the measured yellow halo zone.

#### Ammonia production

Nessler's reagent was used to detect ammonia production by strains. A culture solution containing 10 g of peptone and 5 g of sodium chloride per liter was prepared and divided into 15 ml finger-shaped bottles, and 9.5 ml of culture solution was dispensed into each bottle and then sterilized. A total of 10 μl of each inoculated bacterial solution was tested and incubated at 32°C at 110 rpm for 48 h. Then, 0.5 ml of Nessler's reagent was added, and a change in the color of the liquid bacterial culture from yellow to reddish-brown was observed. The color change was observed, proving that the strain could produce ammonia. The darker the color, the stronger the ammonia production ability it had (Taylor et al., 1988).

#### Nitrogenase activity test

The nitrogenase activity of strains was determined by acetylene reduction assay (ARA), according to the protocol of Hardy et al. (1968). The ethylene with a concentration of 1,000 mg L^−1^ was used as the standard sample. The tested strain was put into a 50 ml headspace flask with 10 ml Ashby culture solution and cultivated for 48 h at 32°C at 150 rpm. A total of 5 ml of gas was taken from the culture flask using a 15 ml syringe, then supplemented with an equal volume of acetylene gas, and kept for 48 h at 32°C at 150 rpm for the detection of the content of ethylene generated by the reduction. The gas chromatograph Agilent 6890M was used to detect nitrogenase activity. The oven temperature, detector temperature, and injector temperature of gas chromatography (GC) were 80, 165, and 180°C, respectively. The ethylene and acetylene retention time was 0.84 min and 0.96 min, respectively. In addition, the flow rates of hydrogen (H_2_) and nitrogen (N_2_) were 40 and 25 ml/min, respectively. The nitrogen-fixing bacteria *Klebsiella variicola* DX120E and *E. roggenkamp*i ED5 were used as control strains, which had been confirmed to have strong nitrogen fixation effects by our previous research.

### Colonization study of *Pseudomonas aeruginosa* DJ06

The strain *P. aeruginosa* DJ06 with a concentration of 1 × 10^6^ CFU ml^−1^ was inoculated into the micropropagated plantlets of sugarcane variety GT11, provided by the Sugarcane Research Institute of Guangxi Academy of Agricultural Sciences, Nanning, Guangxi, China. After being cultured for 3 days, sugarcane roots and stems were collected with sterile scissors, and the surface water was dried with absorbent paper. The sample was treated with 0.1 M of phosphate buffer (pH 7.4) three times for 15 min, then transferred to 1% osmic acid (O_s_O_4_), incubated in the dark incubator at 25°C for 1–2 h, and washed three times. The samples were soaked in alcohol solutions of 30, 50, 70, 80, 90, 95, and 100% for 15 min and then transferred to isoamyl acetate for another 15 min. All the samples were put in the sputtering ion instrument (Hitachi mc1000), sprayed gold for 30 s, and then images were observed with a scanning electron microscope (SEM, Hitachi su8100).

A single colony of strain DJ06, *E. coli* TG1/pPROBE-pTetr-TT-gfp (donor strain), and DH5a/pRK2013 (syncell) were inoculated in the C_2_ medium for 12 h (Supplementary Table 2), centrifuged at 5,000 rpm (5 min), and the supernatant was discarded. Each bacterial liquid (1 ml) was centrifuged three times to remove antibiotics. Donor strains, *E. coli* TG1/pPROBE-TT-pTetr, DJ06, and DH5a/pRK2013, were mixed as 50, 100, and 50 μl (1:2:1), and a 100 μl of the mixture was taken in antibiotic-free C_2_ plates and incubated at 30 °C (6 h). The bacteria were transferred into sterile water and mixed. A total of 100 μl of the mixture was added to the C_2_ plate containing Sm100 and Gm15, and the growth of green fluorescent colonies was observed. The sugarcane GT11 micropropagated plantlets were used to assess the *E. coli* TG1/pprobeptetr TT GFP/DJ06 colonization. The colonization of the DJ06 strain in sugarcane roots and stems was observed by a laser scanning confocal microscope (LSCM) (Lin et al., 2012).

### Genome sequencing and library construction

The genomic DNA of the DJ06 strain was extracted using Wizard^®^ Genomic DNA Purification Kit (Promega), according to the manufacturer's protocol. The purified genomic DNA was quantified by the TBS-380 fluorometer (Turner BioSystems Inc., Sunnyvale, CA). High-quality DNA (OD_260/280_ = 1.8~2.0, >20 μg) was used for further research. Nanopore sequencing and Illumina sequencing platform were used for genome sequencing. The NovaSeq 6000 S4 Reagent Kit 1.5 (300 cycles) was used for Illumina sequencing. The Nanopore library preparation Kit was SQK-LSK11 and Nanopore cell R9.4.1, used to sequence with high-accuracy basecalling (HAC) as the base calling model. Illumina's data evaluation on genome heterozygosity, genome repeatability, genome size, and pollution was performed to ensure the completeness and accuracy of the assembly. The Illumina and nanopore sequencing methods were followed by Gu et al. (2020).

### Genome assembly, gene prediction, and annotation

The original raw data were stored in the fastq format. For accurate assembly, the quality of the original data was checked, and the reads with low sequencing quality, the high proportion of N, and the small length after quality pruning were removed to obtain high-quality clean data. The nanopore data were assembled by Canu software (https://github.com/marbl/canu), and the reads were assembled into contigs and then manually judged into rings to obtain the genome with complete chromosomes. Finally, the assembly results were corrected by Illumina sequencing data. The coding sequence (CDS), tRNA, and rRNA were predicted by Glimmer 3.02, tRNAscan-SE 2.0, and Barrnap software, respectively. The predicted CDS protein functions were annotated from NR, Swiss prot, Pfam, Gene Ontology (GO), Clusters of Orthologous Groups (COG), and Kyoto Encyclopedia of Genes and Genomes (KEGG) databases by using Blast2go 2.0, Diamond 0.8.35, HMMER3.1b2, and other sequence alignment tools (Guo et al., 2021).

### Analysis of genome phylogeny based on average nucleotide identity

The eight similar complete genomes of *P. aeruginosa* DJ06 were selected based on five house-keeping genes (*dnaG, rplB, rpoB, rpsB, smpB*, and *tsf* ) and 16S rRNA gene using the NCBI BLAST search tool (https://blast.ncbi.nlm.nih.gov/Blast.cgi). The ANI was calculated by the OAT software program (https://www.Ezbiocloud.net/tools/orthoani), the results were exhibited in a heatmap using version 3.5.1 gplots 3.0.4 software package, and the cluster analysis was performed by vegan 2.5–6 software (Ciufo et al., 2018).

### Sugarcane seedling and transplanting

To further evaluate the growth-promotion effect of strain *P. aeruginosa* DJ06 on sugarcane, two sugarcane varieties GT11 (requires much fertilizer for growth) and B8 (requires less fertilizer for growth) were tested in the greenhouse. The inoculation concentration of the DJ06 strain was 1 × 10^6^ CFU ml^−1^. The size of the tray for plant culturing was 40 cm in length and 30 cm in width. The red loam soil was used for the experiment. The healthy sugarcane stems were selected and cut into uniform-length segments with bud, which were soaked in hot water at 50°C for 20 min and soaked with 1% carbendazim for 30 min. The treated segments were put into the trays with organic substrates and cultivated at room temperature for approximately 15 days with routine management under greenhouse conditions. When the seedlings grew to 2–3 leaves, those with the same growth status were selected for inoculation and transplanting. The sugarcane seedlings were removed from the tray, and the root surface was washed with sterile water. The roots were soaked in the bacterial solution for approximately 1 h, and the plants were transplanted to the culture pots of a size 32 × 27 cm (H × W). The seedlings treated with sterile water were used as a control. The complete randomized block design (RBD) was applied. There were three groups of inoculation treatment and control for each variety, with 10 pots in each group and one seedling in each pot.

### Evaluation of photosynthetic leaf gas exchange and plant growth parameters

The agronomic traits of sugarcane were investigated 60 days after inoculation. The photosynthesis, transpiration, stomatal conductance, intercellular CO_2_, plant height, and fresh weight were observed in this study. The length from the topsoil to the ring of the top visible dewlap leaf (leaf +1) was measured as plant height. The whole sugarcane plant was removed from the pot, the soil attached to the roots was removed, the roots were rinsed with running water, and the fresh weight was observed after removing the water naturally. The photosynthetic leaf gas exchange was measured by a Li-6800 portable photosynthesis system (Li-COR Biosciences, Lincoln, NE, US) on a sunny day from 9:00 a.m. to 10:30 a.m. (Verma et al., 2020).

### Determination of phytohormones

The content of phytohormones, i.e., indole acetic acid (IAA), gibberellins (GA_3_), and abscisic acid (ABA), of sugarcane varieties GT11 and B8 was tested at 60 days after inoculation by high-performance liquid chromatography (HPLC). A total of 200 mg of samples were ground with liquid nitrogen, then 70–80% methanol solution was added, soaking at 4°C for 12 h, and centrifuged at 12,000 rpm for 10 min at 4°C. In total, 0.5 ml of 70–80% methanol solution was added to the residue after centrifugation and mixed up at 4°C for 2 h before leaching and centrifugation. All the supernatants were combined and evaporated to the one-third volume under reduced pressure at 4°C, and an equal volume of petroleum ether was added. After standing for stratification and repeated extraction and decolorization two to three times, triethylamine was added, and the pH was adjusted to 8.0. After adding cross-linked polyvinylpyrrolidone (PVPP), the mixture was incubated at 150 rpm for 20 min at room temperature (RT). The supernatant was collected after centrifugation, and the pH was adjusted to 3.0. After three extraction times with ethyl acetate, it was evaporated to dryness under reduced pressure at 40°C, added to the mobile phase solution, and vortexed to be fully dissolved. After filtration with a needle filter, the samples were taken and detected by HPLC (WuFeng LC-100, Shanghai, China). The chromatographic conditions were mobile phase A with 100% methanol and B with 0.1% acetic acid aqueous solution (A: B = 55:45). The chromatographic column was C18 reversed-phase chromatographic column (Supelco, United States), in which the injection volume was 20 μl, the column temperature was 30°C, and the size was 150 mm × 4.6 mm × 5 μm. IAA, GA_3_, and ABA retention times were 18.36, 8.36, and 35.00 min, respectively. The ultraviolet detection wavelength was 254 nm.

### Assessment of sugarcane-related enzymatic activities

The enzyme activities in the sugarcane plant were analyzed with an analytical kit 60 days after inoculation. The kits were obtained by Grice Biotechnology Co. Ltd. (Suzhou, China), and the manufacturer's instructions were followed to perform the analysis. In this study, the related oxidative enzymes, such as catalase (CAT, G0105W), peroxidase (POD, G0107W) and superoxide dismutase (SOD, G0101W), the oxidative product, i.e., malondialdehyde (MDA, G0109W), the enzymes related to nitrogen metabolism such as nitrate reductase (NR, G0402W), NADH-glutamate dehydrogenase (NADH-GDH, G0405W) and glutamine synthetase (GS, G0401W), and hydrolases enzymes (endo-1,4-β-D-glucanohydrolase-G0533W and β-1,3-glucanase-G0526W) were examined.

### Data analysis

Agronomic, physiological and biochemical responses were analyzed (ANOVA) by SPSS 20.0 and Excel 2010 software. The difference significance at *p* ≤ 0.05 was used to assess the comparison between the means. Bioinformatics analysis of the complete genome of the DJ06 strain was carried out utilizing the Majorbio I-Sanger Cloud Platform (www.i-sanger.com), which is a free online platform.

## Results

### Isolation and PGP activities of endophytic strains from sugarcane roots

A total of 53 endophytic bacterial strains were isolated using five different media from the roots of the sugarcane variety GT42. Among them, the major 18 (33.97%) strains were isolated from Ashby's mannitol agar, and the most minor five (9.43%) strains from Jensen's agar, and 12 (22.64%), 10 (18.87%), and 8 (15.09%) strains from Pikovskaya's agar, Yeast mannitol agar, and Burk's medium, respectively (Supplementary Figure 1). In addition, 22 strains were found to exhibit diverse PGP activities. The 16S rRNA gene sequencing identification results of these 22 strains are shown in Table 2. The 16S rRNA sequences of the 22 strains were uploaded to the NCBI GenBank database, and accession numbers were MT664177-MT664196 and OP005483-OP005484 (Table 2). Out of them, *in vitro* tests showed that 11 (55%) strains had a positive response to dissolving inorganic phosphate on Pikovskaya's medium plates, and the DJ06 strain showed the most potent activity among them. The results of the siderophore production test found that 13 (59.09%) strains could produce an orange zone in CAS agar plates, and three strains (GB13, GD7, and DJ06) exhibited more vigorous. The growth of the strains with ACC as the sole nitrogen source test showed that 10 (45.45%) strains had positive growth in DF-ACC plates, and two of them (GD13 and DJ06) had more potent activity than other strains. In addition, all the strains had potential ammonia production capacity, and GA16, GA20, GB26, GD3, GD6, GD7, GD13, and DJ06 exhibited more robust activity. Overall, the results showed that strain *P. aeruginosa* DJ06 had more prominent PGP activities than other strains (Table 2). In addition, the nitrogen-fixing capacity of DJ06 was tested through the ARA method. The result showed that the nitrogenase activity reached 31.27 nmol C_2_H_4_ mg protein h^−1^, which was higher at 3.29% and lower at 4.64% than the control strains of *Enterobacter roggenkampii* ED5 and *Klebsiella variicola* DX120E, respectively, indicating that the DJ06 strain has strong nitrogen fixation potential (Table 3).

**Table 2:** PGP activities of selected endophytic strains from the sugarcane roots.

**Isolations**	**Most similar strain**	**Similarity (%)**	**NCBI accession No**.	**Phosphate**	**Siderophore**	**DF-ACC**	**HCN**	**Ammonia**
GA8	*Enterobacter cloacae*	99.93%	MT664177	+	+	-	+	+
GA16	*Enterobacter* sp.	99.93%	MT664178	+	+	+	-	+++
GA20	*Enterobacter* sp	99.93%	MT664179	-	-	-	+	+++
GB2	*Enterobacter* sp.	99.93%	MT664180	+	-	+	++	+
GB3	*Leclercia adecarboxylata*	99.79%	MT664194	+	-	-	-	++
GB8	*Enterobacter* sp	99.86%	MT664181	+	-	+	-	++
GB13	*Enterobacter* sp.	99.86%	MT664182	+	+++	+	-	++
GB21	*Enterobacter aerogenes*	99.79%	MT664183	+	-	+	+	++
GB22	*Enterobacter asburiae*	99.18%	MT664184	-	-	-	-	++
GB25	*Enterobacter aerogenes*	99.66%	MT664185	-	+	-	+	++
GB26	Uncultured *Enterobacter* sp.	99.93%	MT664186	-	-	-	++	+++
GB27	*Bacterium strain*	99.93%	MT664195	+	-	-	-	+
GC16	*Enterobacter cloacae*	99.93%	MT664187	+	+	-	+	++
GD3	*Enterobacter oryzae*	99.86%	MT664188	-	-	+	+	+++
GD6	Uncultured *Enterobacter* sp.	99.79%	MT664189	-	-	-	-	+++
GD7	*Enterobacter oryzae*	99.79%	MT664190	-	+++	-	+	+++
GD9	*Enterobacter* sp.	99.79%	MT664191	-	-	-	+	+
GD12	*Enterobacter* sp.	100%	MT664192	-	+	-	+	++
GD13	*Kosakonia oryzae*	99.93%	MT664196	+	+	++	+	+++
GD16	*Enterobacter* sp.	99.86%	MT664193	+	+	+	-	++
DJ06	*Pseudomonas aeruginosa*	99.45%	OP005483	++	+++	+++	+++	+++
DJ08	Uncultured bacterium	99.65%	OP005484	+	+	+	+	++

(-), No Activity; (+++), Strong Activity; (++), Moderate Activity; (+), Low activity.

**Table 3:** The nitrogenase activity in the DJ06 strain in comparison with the other two strains.

**Strains**	**ARA (nmoL C_2_H_4_ mg protein h^−1^)**
*Pseudomonas aeruginosa* DJ06	31.27 ± 0.23^b^
*Enterobacter roggenkampii* ED5	30.24 ± 0.19^c^
*Klebsiella variicola* DX120E	32.72 ± 0.73^a^

All data points are presented as mean ± SE (*n* = 3). Different lowercase letters indicate a significant difference at *p* < 0.05 according to DMRT (Duncan's Multiple Range Test).

### Colonization of GFP-tagged endophytic DJ06 in sugarcane tissues

**Figure 1:**
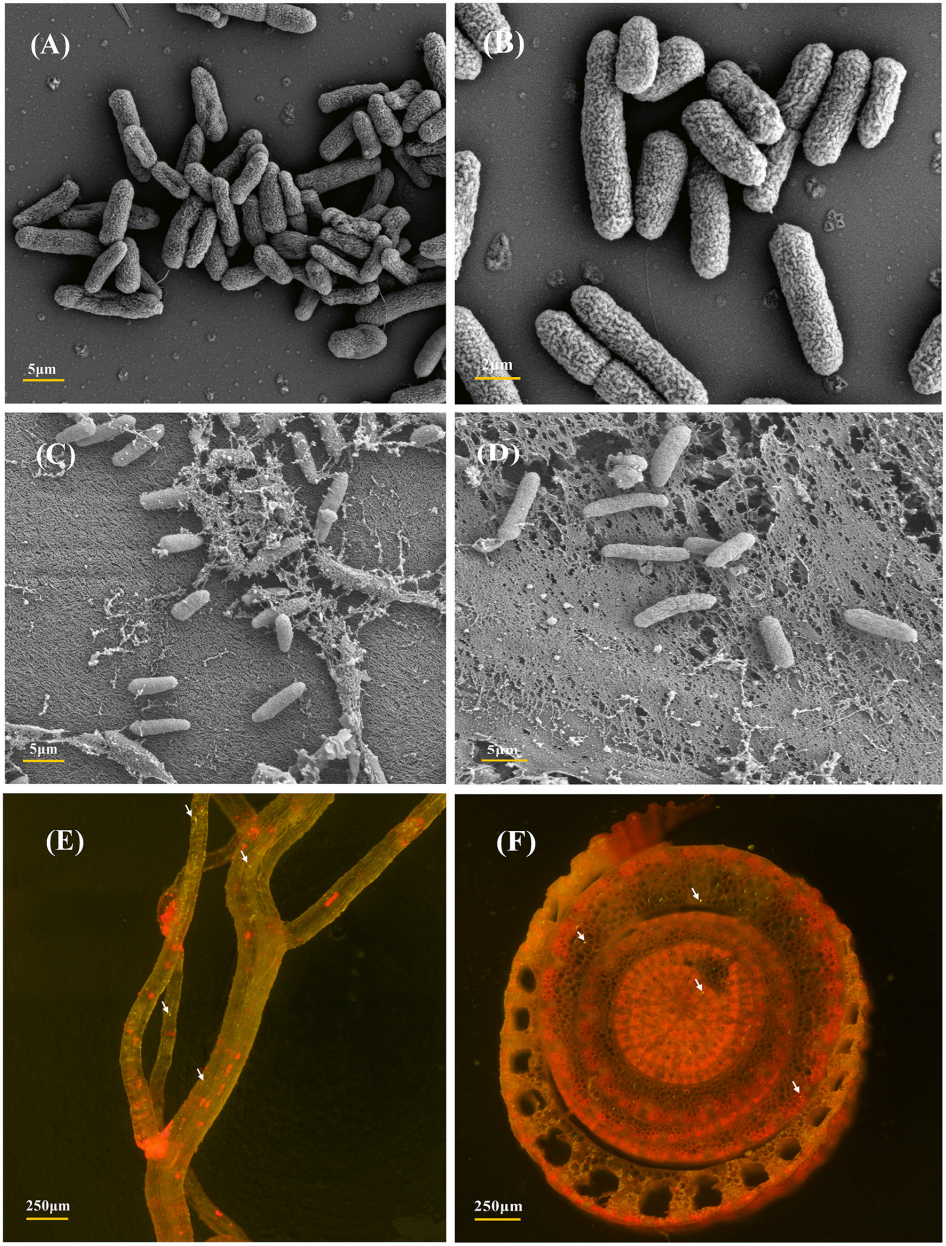
SEM and CLSM micrographs of the most efficient endophytic *Pseudomonas aeruginosa* DJ06 strain and its colonization in sugarcane plant parts at the root and stem regions. **(A, B)** Are the SEM images showing the morphology of the DJ06 strain, **(C, D)** are the colonization images obtained after the inoculation of the DJ06 strain in sugarcane root and stem, **(E, F)** show the CLSM micrographs of GFP-tagged endophytic DJ06 strain in sugarcane root and stem. Green fluorescence within the root and stem tissues indicated by white arrows indicates that the DJ06 strain has colonized in the tissue.

The strain *P. aeruginosa* DJ06, which has various potential PGP characteristics, was used to analyze the colonization in sugarcane tissue. The sugarcane root colonization and colony morphology of the DJ06 strain were observed by SEM and CLSM (Figure 1). Colonization analysis was fundamental for further study of the interaction mechanism between the DJ06 strain and the sugarcane plant. The morphology of the DJ06 strain and colonization in plant roots and stems is presented in Figures 1A–D. It was found that the DJ06 strain could successfully colonize sugarcane tissue observed using SEM. In addition, the GFP-tagged DJ06 was observed by CLSM after inoculation in sugarcane seedlings for 3 days. The results showed that the roots and stems of the seedlings had green fluorescent bacteria, which proved that the DJ06 strain had colonized the sugarcane tissues (Figures 1E, F).

### Genomic properties of the DJ06 strain

**Figure 2:**
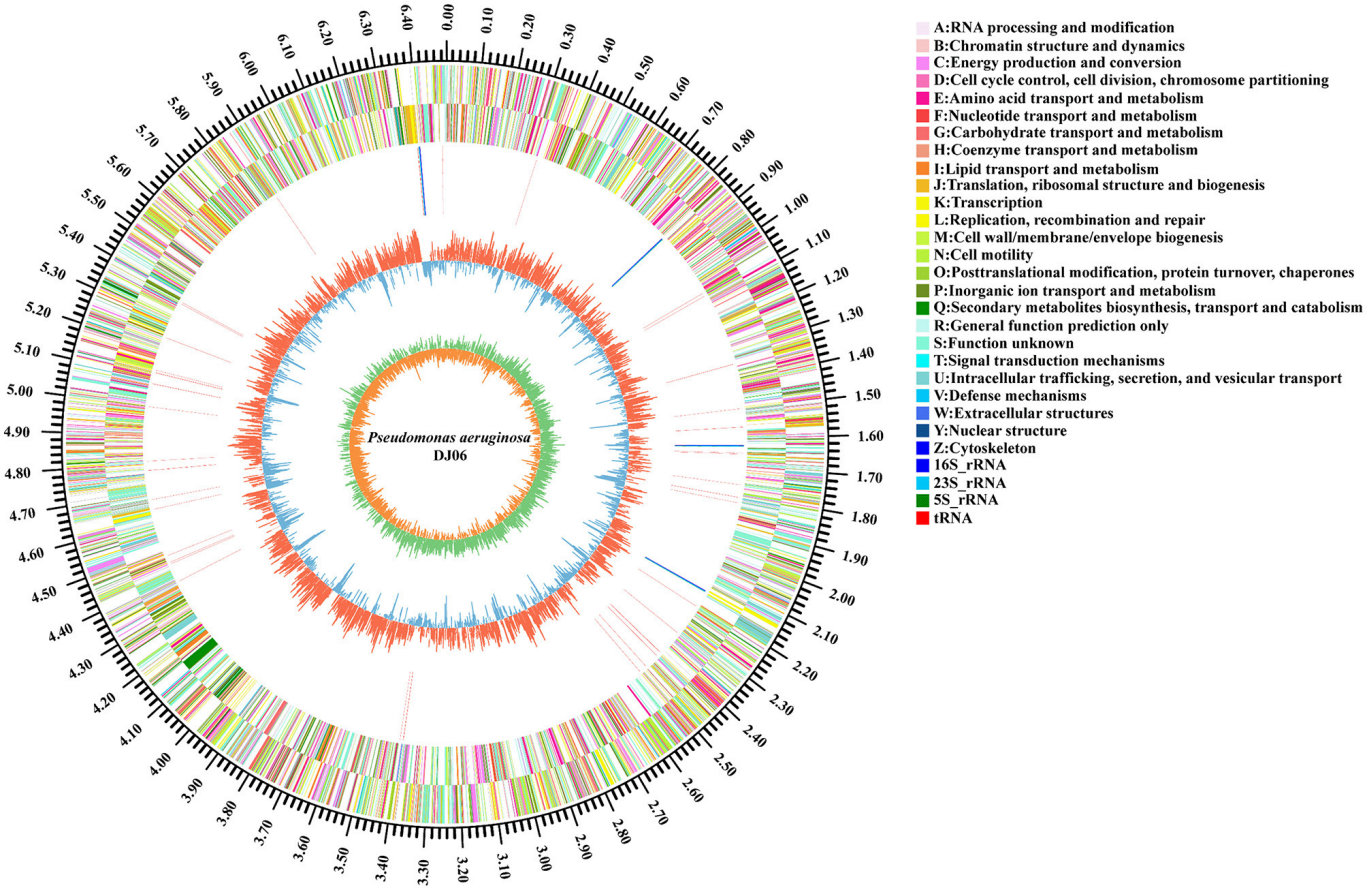
Circular representation of chromosome of endophytic bacteria *Pseudomonas aeruginosa* DJ06 strain isolated from sugarcane root. The inner and innermost rings display the GC content and skew. Circles 1–2 show the functional classification of the CDS genes in the chromosome with the colors of the COG database, and in circles 3, different colors indicate different RNA types.

The general properties of the genome of endophytic strain *P. aeruginosa* DJ06 are shown in Table 4, with a 64,90,034 bp of circular chromosome (Figure 2). The average G+C content in the genome was 66.34% including 5,912 CDSs. In addition, the *P. aeruginosa* DJ06 genome had 12 rRNA genes with 5S, 16S, and 23S, six genomic islands, eight CRISPR, and three prophages, respectively. The CDSs were annotated in GO, COG, and KEGG databases, which were 4,375, 5,251, and 3,246, respectively, involving multiple biological processes (Supplementary Figures 2–4). The complete sequence of the strain *P. aeruginosa* DJ06 has been submitted at the NCBI/GeneBank with accession number CP080511.

**Table 4:** Genome characteristic of endophytic strain *Pseudomonas aeruginosa* DJ06.

**Characteristics**	**Value**
Genome size (bp)	6,490,034 bp
GC content (%)	66.34%
tRNA	65
rRNA (5S, 16S, 23S)	12 (4, 4, 4)
Protein-coding genes (CDS)	5,912
Genomic islands	6
CRISPR	8
Prophge	3
Genes assigned to NR	5,909
Genes assigned to Swiss-Prot	4,504
Genes assigned to COG	5,251
Genes assigned to KEGG	3,246
Genes assigned to GO	4,375
Genes assigned to Pfam	5,178

### Genome-based phylogeny of the DJ06 strain

**Figure 3:**
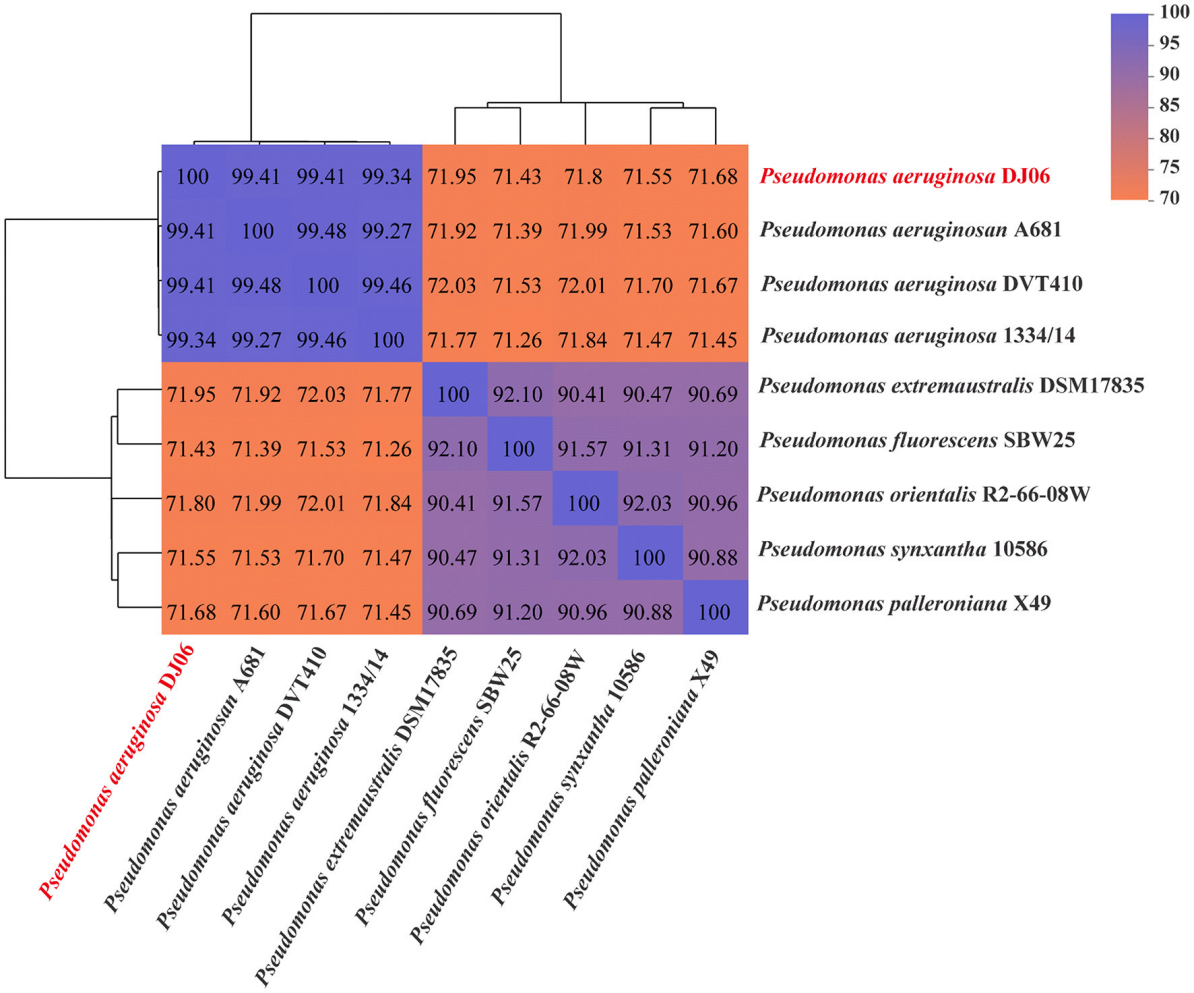
Phylogenetic analysis of *Pseudomonas aeruginosa* DJ06 genome based on ANI.

The ANI results showed that the genome of DJ06 presented 99.41% ANI to *P. aeruginosa* A681 and *P. aeruginosa* AVT410 and 94.34% ANI to *P. aeruginosa* 1334/14, respectively. The ANI values of the DJ06 strain and other strains were less than 95%; the highest value was 71.95% for *P. extremaustralis* DSM17835, and the lowest was 71.43% for *P*. *fluorescens* SBW25. These ANI results indicated that the DJ06 strain belongs to *P. aeruginosa*, which was also confirmed by cluster analysis (Figure 3).

### Potential PGP-related genes in endophytic strain DJ06 genome

Some essential PGP-related genes were predicted in the genome of the DJ06 strain, such as nitrogen fixation (*iscU*), ammonia assimilation (*gltB*), nitrosative stress (*fpr, hmp, gbcB*, and *vanB*), siderophore (*tonB, fabY, fepA*, and *fhuE*), IAA production (*trpCEGS*), phosphate metabolism (*pit, pstABCS*, and *PhoABDUR*), hydrolase (*bglXB, folE, folE2, ribA, and ribBA*), HCN (*hcnABC*), phenazine (*phzA_B, phzF*), chemotaxis (*cheABDVRWAZ, pilIJK, aer*, and *mcp*), sulfur metabolism (*cysABCDEG, HI, JK, SP, UWYZ*, and *cysNC*), and biofilm formation (*flgABCDEFGHIJKLMN, motAB*) (Table 5).

**Table 5:** Genes associated with PGP traits in *Pseudomonas aeruginosa* DJ06 genome.

**PGP activities description**	**Gene name**	**Gene annotation**	**E.C. number**	**Chromosome location**
Nitrogen fixation	*-*	Nitrogen fixation protein FixS OS	-	3119172–3118963, -
	*-*	Nitrogen fixation protein FixS OS	-	3123549–3122134, -
	*iscU*	nitrogen fixation protein NifU and related proteins	-	5861277–5860891, -
	*-*	Nitrogen fixation protein FixG OS	-	6223989–6225419, +
Ammonia assimilation	*gltB*	Glutamate biosynthetic process; ammonia assimilation cycle	1.4.1.13	1212098–1216543, +
	*-*	Glutamate biosynthetic process;;ammonia assimilation cycle	-	5621876–5623486, +
Nitrosative stress	*fpr*	Is involved in NO detoxification in an aerobic process	1.18.1.2 1.19.1.1	1720673–1721449, +
	*-*	Response to nitrosative stress	-	4540178–4539921, -
	*hmp*	Response to nitrosative stress	1.14.12.17	4541414–4540233, -
	*fpr*	Is involved in NO detoxification in an aerobic process	1.18.1.2 1.19.1.1	5402930–5402154, -
	*gbcB*	Is involved in NO detoxification in an aerobic process	-	795365–794265, -
	*vanB*	Is involved in NO detoxification in an aerobic process		1381843–1380890, -
	*-*	Ferredoxin reductase	-	1399767–1400867, +
ACC deaminase	*-*	1-aminocyclopropane-1-carboxylate deaminase	3.5.99.7	5071988–5071089, -
Siderophore	*-*	TonB-dependent siderophore receptor	-	88310–90703, +
	*-*	tonB-dependent siderophore receptor	-	134496–132304, -
	*tonB*	Siderophore transmembrane transporter activity	-	389670–388858, -
	*-*	Siderophore uptake transmembrane transporter activity	-	446014–443627, -
	*tonB*	Siderophore transmembrane transporter activity	-	656442–657473, -
	*fabY*	Siderophore biosynthetic process; cytosol	2.3.1.180	1058808–1056904, -
	*-*	Siderophore uptake transmembrane transporter activity	-	1456126–1458252, +
	*fur*	Ferric iron uptake transcriptional regulator	-	1542620–1543024, +
	*tbpA*	Siderophore transmembrane transport	-	1605480–1603186, -
	*-*	TonB-dependent receptor	-	1651522–1649294, -
	*fiu*	TonB-dependent siderophore receptor	-	1858562–1860823, +
	*-*	siderophore transmembrane transport	-	2267255–2265192, -
	*fepA*	TonB-dependent siderophore receptor	-	2442960–2445188, +
	*fiu*	TonB-dependent siderophore receptor	-	2862829–2865027, +
	*-*	TonB-dependent receptor	-	2906036–2908477, +
	*-*	TonB-dependent siderophore receptor	-	3525154–3522740, -
	*fecI*	Siderophore transport	-	3526815–3526309, -
	*-*	Hypothetical protein	-	3661863–3662819, +
	*fhuE*	TonB-dependent siderophore receptor	-	4124341–4121894, -
	*mbtH*	Chain X, Hypothetical Protein Pa2412	-	4160575–4160357, -
	*-*	Outer membrane receptor protein, mostly Fe transport	-	4258973–4256511, -
	*fepA*	Siderophore enterobactin receptor PfeA	-	4567882–4570122, +
	*pfeE*	Ferric enterobactin esterase PfeE	3.1.1.108	4570142–4571056, +
	*-*	TonB-dependent receptor	-	4868884–4871040, +
	*fecA*	TonB-dependent receptor family protein	-	5251782–5249617, -
	*-*	TonB-dependent copper receptor	-	5840255–5838096, -
	*fecA*	TonB-dependent receptor family protein	-	5953771–5956125, +
	*-*	TonB-dependent receptor	-	6256838–6254754, -
	*fhuE*	TonB-dependent siderophore receptor	-	6273393–6275801, +
	*-*	RhtX/FptX family siderophore transporter	-	6332484–6331240, -
*fhuE*	TonB-dependent siderophore receptor	-	6336427–6334265, -
IAA production	*trpC*	Indole-3-glycerol phosphate synthase TrpC	4.1.1.48	6420339–6419503, -
	*trpS*	Tryptophanyl-trna synthetase	6.1.1.2	1970955–1972301, +
	*trpE*	Anthranilate synthase component I	4.1.3.27	2499727–2501298, +
	*trpG*	Anthranilate synthase component II	4.1.3.27	2501276–2501878, +
	*trpG*	Aminodeoxychorismate/anthranilate synthase component II	4.1.3.27	6421992–6421387, -
	*trpE*	Anthranilate synthase component I	4.1.3.27	6440605–6439127, -
Auxin biosynthesis	*-*	Auxin Efflux Carrier	-	727207–726263, -
	*-*	Auxin Efflux Carrier	-	3320366–3321253, +
	*-*	Auxin Efflux Carrier	-	402421–401540, -
Phosphate metabolism	*pit*	Inorganic phosphate transporter	-	2129593–2128124, -
	*pstS*	Phosphate-binding protein PstS OS	-	4352689–4354074, +
	*pstS*	Phosphate ABC transporter substrate-binding protein	-	843290–844261, +
	*pstC*	Phosphate ABC transporter permease	-	844432–846717, +
	*pstA*	Phosphate ABC transporter permease PstA	-	846737–848413, +
	*pstB*	Phosphate ABC transporter ATP-binding protein	7.3.2.1	848429–849262, +
	*phoU*	Chain A, Phosphate-specific Transport System Accessory Protein Phou Homolog	-	849358–850086, +
	*phoR*	Phosphate regulon sensor histidine kinase PhoR	-	854931–853600, -
	*phoB*	Phosphate regulon transcriptional regulator PhoB	-	855693–855004, -
	*phoA*	MULTISPECIES: alkaline phosphatase	3.1.3.1	5283151–5284581, +
	*phoD*	Alkaline phosphatase	3.1.3.1	5966444–5964882, -
Hydrolase	*-*	Chitinase	-	3999213–3997762, -
	*-*	Chitinase	-	4393909–4393280, -
	*-*	Chitinase	-	4699259–4698630, -
	*-*	Glycoside hydrolase family 19 protein	-	6423582–6422953, -
	*-*	Cellulase activity	-	3829462–3830538, +
	*pslG*	Cellulase family glycosylhydrolase	-	3928966–3930294, +
	*bglB*	Family 1 glycosylhydrolase	3.2.1.21	1352806–1351265, -
	*bglX*	Beta-glucosidase BglX	3.2.1.21	3311378–3309084, -
	*folE2*	GTP cyclohydrolase IB	3.5.4.16	650698–649802, -
	*folE*	GTP cyclohydrolase 1 2	3.5.4.16	3268888–3268343, -
	*folE*	Dihydromonapterin reductase	3.5.4.16	5443637–5444197, +
	*ribA*	MULTISPECIES: GTP cyclohydrolase II	3.5.4.25	6135458–6134841, -
	*ribBA*	GTP cyclohydrolase II	4.1.99.12 3.5.4.25	6140653–6139556, -
HCN	*hcnA*	Cyanide-forming glycine dehydrogenase subunit HcnA	1.4.99.5	3843114–3843428, +
	*hcnB*	Cyanide-forming glycine dehydrogenase subunit HcnB	1.4.99.5	3843425–3844819, +
	*hcnC*	Cyanide-forming glycine dehydrogenase subunit HcnC	1.4.99.5	3844822–3846075, +
Phenazine	*phzA_B*	Phenazine biosynthesis protein PhzA 2	-	3511140–3511628, +
	*phzA_B*	MULTISPECIES: phenazine biosynthesis protein phzB 2	-	3511664–3512152, +
	*phzE*	Phenazine-specific anthranilate synthase component I	2.6.1.86	3514010–3515893, +
	*phzF*	PhzF family phenazine biosynthesis protein	5.3.3.17	3515907–3516743, +
	*phzA_B*	Phenazine biosynthesis protein	-	6323470–6323958, +
	*phzA_B*	Phenazine biosynthesis protein	-	6323988–6324476, +
	*phzF*	PhzF family phenazine biosynthesis protein	5.3.3.17	6328241–6329077, +
Chemotaxis	*cheY*	Chemotaxis protein CheY	-	3019761–3020135, +
	*cheZ*	Chemotaxis protein CheZ	-	3020155–3020943, +
	*cheA*	Chemotaxis protein CheA	-	3021144–3023405, +
	*cheB*	CheB methylesterase	-	3023459–3024565, +
	*cheW*	Purine-binding chemotaxis protein CheW	-	3027272–3028162, +
	*cheW*	Chemotaxis protein CheW	-	3028208–3028687, +
	*cheR*	Chemotaxis protein methyltransferase 1 OS	2.1.1.80	5354447–5353623, -
	*cheV*	Chemotaxis protein CheV1 OS	-	5355456–5354524, -
	*cheY*	Chemotaxis protein CheY OS	-	412592–412957, -
	*cheA*	Chemotaxis protein CheA	-	412985–414913, +
	*cheW*	Chemotaxis protein CheW OS	-	414900–415385, +
	*cheR*	Chemotaxis protein methyltransferase 2	2.1.1.80	417530–418372, +
	*cheD*	Chemoreceptor glutamine deamidase CheD	3.5.1.44	418378–418980, +
	*cheB*	Chemotaxis response regulator protein	3.5.1.44	419000–420049, +
	*aer*	Aerotaxis receptor Aer	-	3134157–3132592, -
	*mcp*	Methyl-accepting chemotaxis protein	-	411222–412394, +
	*pilK*	Chemotaxis protein methyltransferase OS	-	166216–165341, +
	*pilJ*	Methyl-accepting chemotaxis protein (MCP)	-	168325–166277, -
	*pilI*	Chemotaxis protein CheW	-	168946–168410, -
Sulfur metabolism	*cysW*	Sulfate ABC transporter permease subunit CysW	-	304421–305290, +
	*cysA*	Sulfate ABC transporter ATP-binding protein	7.3.2.3	305294–306283, +
	*cysU*	Sulfate ABC transporter permease subunit CysT	-	303592–304410, +
	*cysJ*	PepSY domain-containing protein	1.8.1.2	1861039–1863591, +
	*cysD*	Sulfate adenylyltransferase subunit CysD	2.7.7.4	1966763–1967680, +
	*cysNC*	Sulfate adenylyltransferase subunit CysN	2.7.7.4 2.7.1.25	1967692–1969593, +
	*cysZ*	Sulfate transporter CysZ OS	-	2349639–2348899, -
	*cysK*	PLP-dependent cysteine synthase family protein	2.5.1.47	2564594–2565691, +
	*cysC*	Adenylyl-sulfate kinase	2.7.1.25	2950058–2949468, -
	*cysP*	Sulfate ABC transporter substrate-binding protein	-	3055525–3054527, -
	*cysB*	HTH-type transcriptional regulator CysB OS	-	3337225–3338199, +
	*cysH*	Phosphoadenylyl-sulfate reductase	1.8.4.8 1.8.4.10	3339402–3338668, -
	*cysS*	Ysteine–tRNA ligase	6.1.1.16	3387721–3389103, +
	*cysI*	Nitrite/sulfite reductase	1.8.1.2	3439778–3438120, -
	*cysK*	PLP-dependent cysteine	2.5.1.47	3751289–3752206, +
	*cysG*	Uroporphyrinogen-III C-methyltransferase	2.1.1.107	4479697–4478300, -
	*cysE*	Serine O-acetyltransferase	2.3.1.30	5863969–5863193, -
	*cysI*	Nitrite/sulfite reductase	1.8.1.2	6223592–6221919, -
Biofilm formation	*flgB*	flagellar basal body rod protein FlgB	-	2580028–2580435, +
	*flgC*	flagellar basal body rod protein FlgC	-	2580441–2580881, +
	*flgD*	flagellar hook assembly protein FlgD	-	2580894–2581607, +
	*flgE*	Flagellar hook protein FlgE	-	2581635–2583023, +
	*flgF*	Flagellar basal-body rod protein FlgF	-	2583241–2583990, +
	*flgG*	Flagellar basal-body rod protein FlgG	-	2584037–2584822, +
	*flgH*	Flagellar L-ring protein precursor FlgH	-	2584868–2585563, +
	*flgI*	Flagellar basal body P-ring protein FlgI	-	2585575–2586684, +
	*flgJ*	Flagellar assembly peptidoglycan hydrolase FlgJ	-	2586695–2587897, +
	*flgK*	Flagellar hook-associated protein FlgK	-	2587916–2589964, +
	*flgL*	Flagellar hook-associated protein FlgL	-	2590003–2591304, +
	*flgA*	Flagellar biosynthesis protein FlgA	-	5355549–5356286, +
	*flgM*	Flagellar biosynthesis anti-sigma factor FlgM	-	5356431–5356754, +
	*flgN*	Flagellar protein FlgN	-	5356809–5357279, +
	*motA*	Flagellar motor protein MotA	-	1324368–1325219, +
	*motB*	Flagellar motor protein MotB	-	1325239–1326282, +
	*motA*	flagellar motor protein	-	3024654–3025394, +
	*motB*	flagellar motor protein MotD	-	3025407–3026297, +

**Figure 4:**
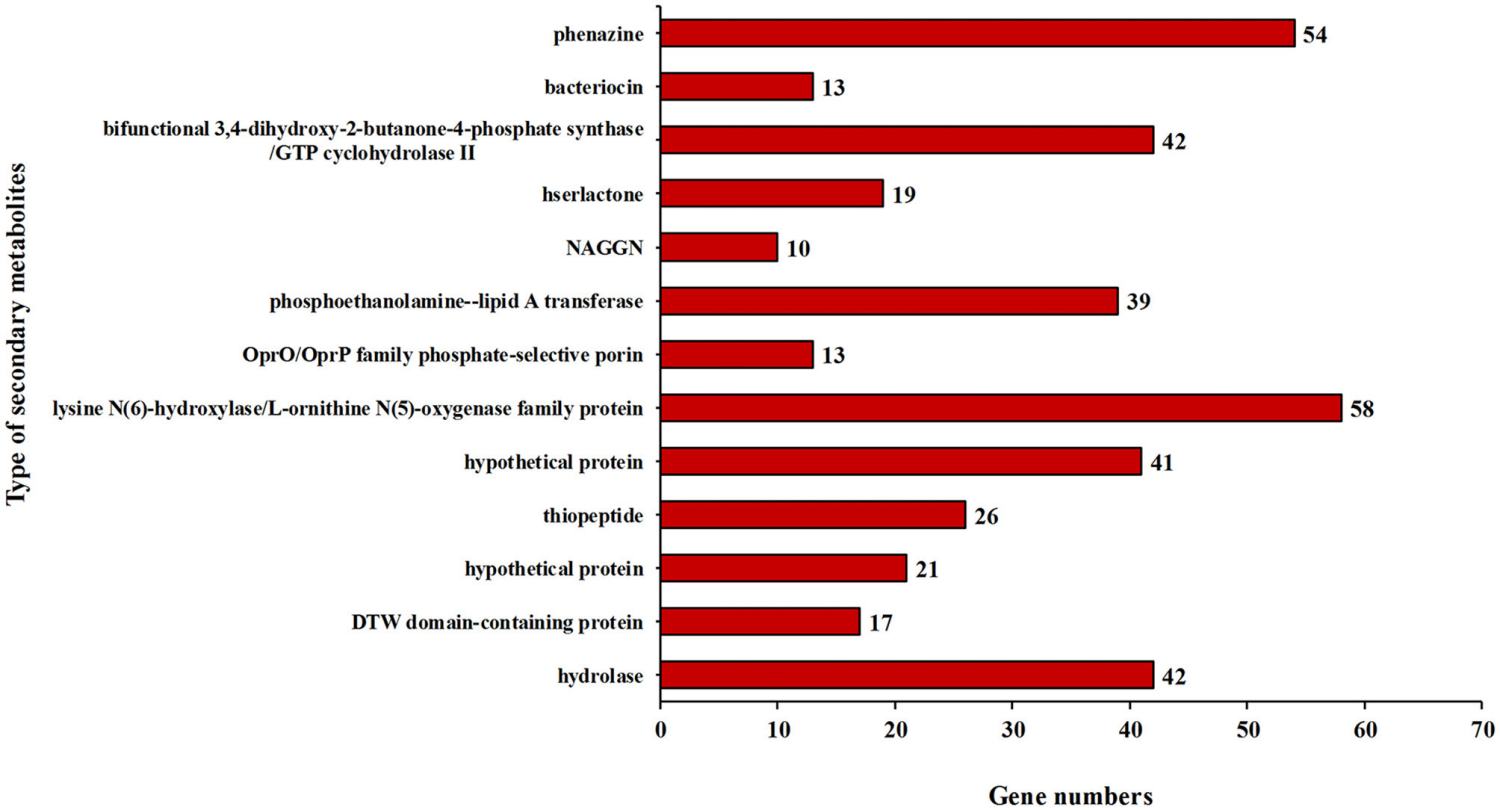
Secondary metabolic gene clusters in the genome of strain *Pseudomonas aeruginosa* DJ06.

In addition, some key genes in the DJ06 strain genome involved in plant resistance to abiotic stresses, for example, those coding for a cold-shock protein (*cspA*), heat shock proteins (*htpRX, hslJR*, and *ibpA*), magnesium transport (*corAC*), copper homeostasis (*copABZ, cusRS*), zinc homeostasis (*znuABC*), chromium homeostasis (*czcACD*), and drought resistance (*nhaB, kdpABCDE, proABCSVWX, betABT*, and *trkAH*), were categorized (Table 6). By using the software of antiSMSAH 4.0.2, the genome was predicted to contain various secondary metabolites such as phenazine, bacteriocin, and bifunctional 3,4-dihydroxy-2-butanone-4-phosphate synthase/GTP cyclohydrolase II (Figure 4).

**Table 6:** Genes involved in different abiotic stresses in *Pseudomonas aeruginosa* DJ06 genome.

**Activities description**	**Gene name**	**Gene annotation**	**E.C. number**	**Chromosome location**
Cold-shock protein	*cspA*	Cold-shock protein	-	2472057–2471443, -
	*cspA*	Cold-shock protein	-	5247298–5247507, +
	*cspA*	Cold-shock protein	-	106158–105949, -
	*cspA*	Cold-shock protein	-	2675545–2675754, +
	*cspA*	Cold shock domain protein CspD	-	4491483–4491755, +
Heat shock proteins	*htpX*	Heat shock protein HtpX	3.4.24.-	4785931–4786890, +
	*hslR*	Heat-shock protein	-	1034089–1034490, +
	*ibpA*	16 kDa heat shock protein B OS	-	5112206–5111757, -
	*hslJ*	Heat shock protein HslJ	-	2436184–2435777, -
Magnesium transport	*corA*	Magnesium transport protein CorA	-	947776–948807, +
	*corC*	HlyC/CorC family transporter	-	6048076–6048915, +
Copper homeostasis	*copB*	P-type Cu2+ transporter	7.2.2.9	3121586–3119151,-
	*copZ*	Heavy-metal-associated domain-containing protein		5518289–5518095, -
	*copA*	Copper-translocating P-type ATPase	7.2.2.8	5974427–5976805, +
	*cusS*	Heavy metal sensor histidine kinase CusS	2.7.13.3	1404894–1403503, -
	*cusR*	Copper resistance phosphate regulon response regulator CusR	-	1405670–1404873, -
	*cusR*	Heavy metal response regulator transcription factor	-	3001305–3001994, -
	*cusS*	Heavy metal sensor histidine kinase CusS	2.7.13.3	3002002–3003417, +
	*cusR*	Heavy metal response regulator transcription factor	2.7.13.3	4317562–4318236, +
	*cusR*	Heavy metal sensor histidine kinase CusS	-	4760863–4761543, +
	*cusS*	Heavy metal response regulator transcription factor	2.7.13.3	4761540–4762871, +
Zinc homeostasis	*znuB*	High-affinity zinc uptake system membrane protein znuB	-	688886–688098, -
	*znuC*	Zinc ABC transporter ATP-binding protein ZnuC	7.2.2.-	689688–688879, -
	*znuA*	Zinc ABC transporter substrate-binding protein	-	690261–691184, +
	*znuA*	Zinc ABC transporter substrate-binding protein	-	4155971–4156924, +
	*znuC*	Zinc ABC transporter substrate-binding protein	7.2.2.-	4156921–4157676, +
	*znuB*	Zinc transport system permease protein	-	4157673–4158578,+
Chromium homeostasis	*copB*	Multispecies: cadmium-translocating P-type ATPase	7.2.2.9	3121586–3119151, -
	*-*	Cadmium-translocating P-type ATPase	-	4198337–4196352, -
	*czcA*	Cobalt-zinc-cadmium resistance protein CzcA OS	-	4314233–4311078, -
	*czcC*	Cobalt-zinc-cadmium resistance protein CzcC OS	-	4317049–4315763, -
	*czcD*	Cobalt-zinc-cadmium efflux system protein	-	179617–180516, +
Drought resistance	*nhaB*	Na(+)/H(+) antiporter NhaB OS	-	3422913–3421411, -
	*kdpA*	Potassium-transporting ATPase subunit KdpA	-	3210235–3211929, +
	*kdpB*	Potassium-transporting ATPase subunit KdpB	7.2.2.6	3211941–3214013, +
	*kdpC*	Potassium-transporting ATPase subunit KdpC	-	3214067–3214618, +
	*kdpD*	Two-component system, OmpR family, sensor histidine kinase KdpD	2.7.13.3	3214726–3217383, +
	*-*	Citrate-Mg2+:H+ or citrate-Ca2+:H+ symporter, CitMHS family	-	725624–724320, -
	*GDT1*	Ca2+/H+ antiporter, TMEM165/GDT1 family	-	1697520–1696942, -
	*kdpE*	Response regulator	-	3217484–3218176, +
	*proC*	Pyrroline-5-carboxylate reductase	1.5.1.2	183803–184624, +
	*proX*	Glycine betaine/proline transport system substrate-binding protein	-	584983–585906, +
	*proX*	Glycine betaine/proline transport system substrate-binding protein	-	815673–816611, +
	*proX*	Glycine betaine/proline transport system substrate-binding protein	-	826054–826992, +
	*proW*	Glycine betaine/proline transport system substrate-binding protein		827034–827873, +
	*proV*	Glycine betaine/proline transport system ATP-binding protein	7.6.2.9	827877–829055, +
	*proB*	Glutamate 5-kinase	2.7.2.11	1779627–1780745, +
	*proS*	Chain A, Proline–tRNA ligase	6.1.1.15	2468134–2466419, -
	*proA*	Glutamate-5-semialdehyde dehydrogenase	1.2.1.41	6071938–6070673, -
	*betA*	Choline dehydrogenase	1.1.99.1	3771488–3773125, +
	*betT*	Choline/glycine/proline betaine transport protein	-	5994121–5996082, +
	*betB*	Betaine-aldehyde dehydrogenase	1.2.1.8	831810–833282, +
	*trkA*	Trk system potassium transporter TrkA	-	599227–600600, +
	*trkH*	Potassium uptake protein TrkH	-	5192548–5194002, +

### Plant growth parameters

**Figure 5:**
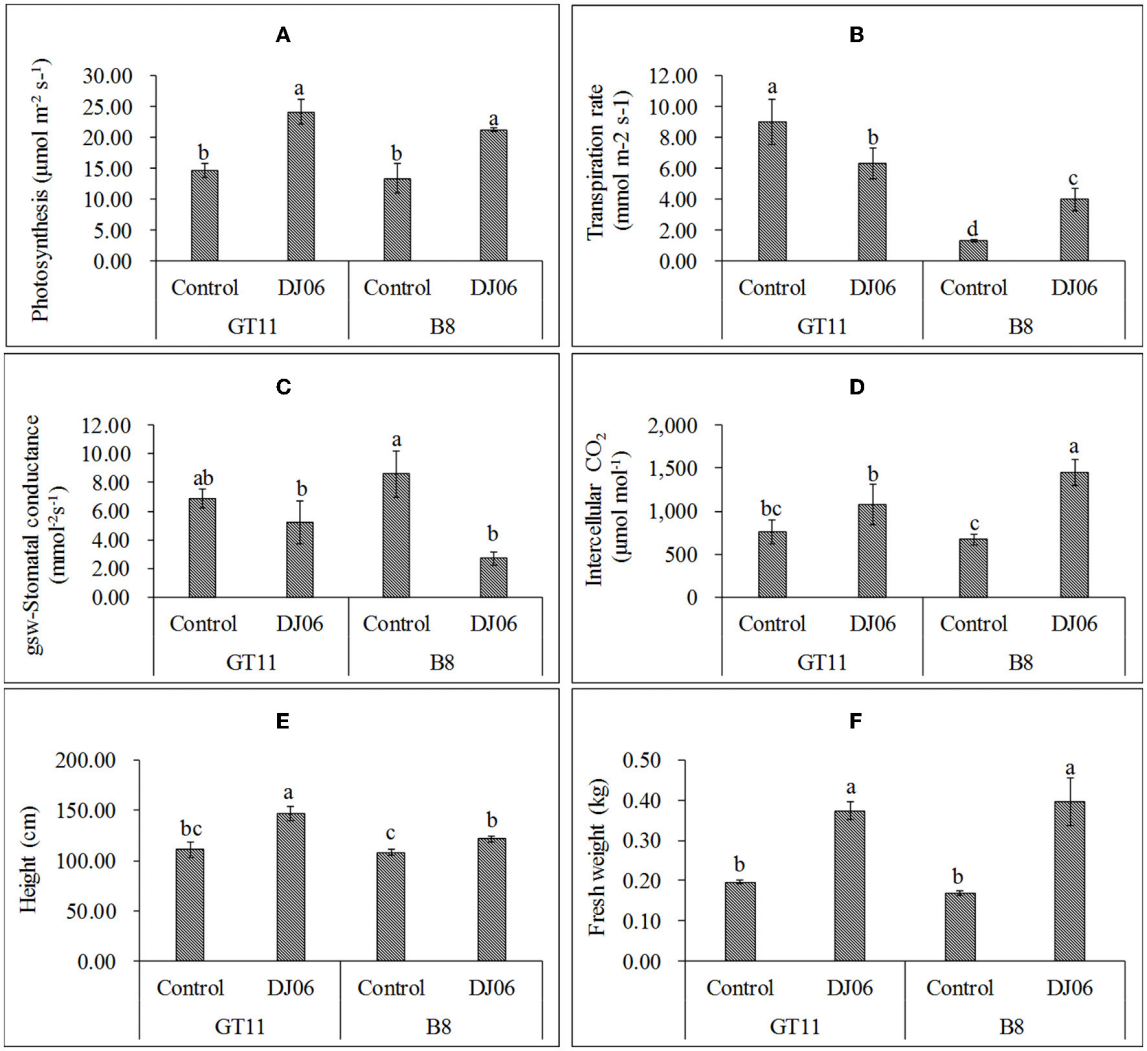
The agronomic growth parameters in sugarcane varieties GT11 and B8 60 days after inoculation with *Pseudomonas aeruginosa* DJ06 compared with the control. **(A)** Photosynthesis; **(B)** transpiration rate; **(C)** stomatal conductance; **(D)** intercellular CO_2_; **(E)** height; and **(F)** fresh weight. The same letter indicated that no significant difference was detected at Duncan's multiple range test, *P* ≤ 0.05 (*n* = 3).

In the present study, the DJ06 strain had a growth-promoting effect on sugarcane varieties GT11 and B8 at 60 days after inoculation compared with the control (Supplementary Figure 5). The photosynthetic rate in the two varieties was significantly increased by 64.35 and 59.18%, respectively. The transpiration rate in variety GT11 was significantly decreased, whereas that in B8 was enhanced nearly 2-fold. The stomatal conductance in GT11 and B8 was reduced by 24.27 and 68.33%, respectively. However, the intercellular CO_2_ in GT11 and B8 was enhanced by 41.69 and 116.22%, respectively. In addition, the plant height in GT11 and B8 increased significantly by 32.43 and 12.66 %, and the fresh weight was improved by 89.87 and 135.71% as compared with the control (Figure 5).

### Plant endogenous hormones

**Figure 6:**
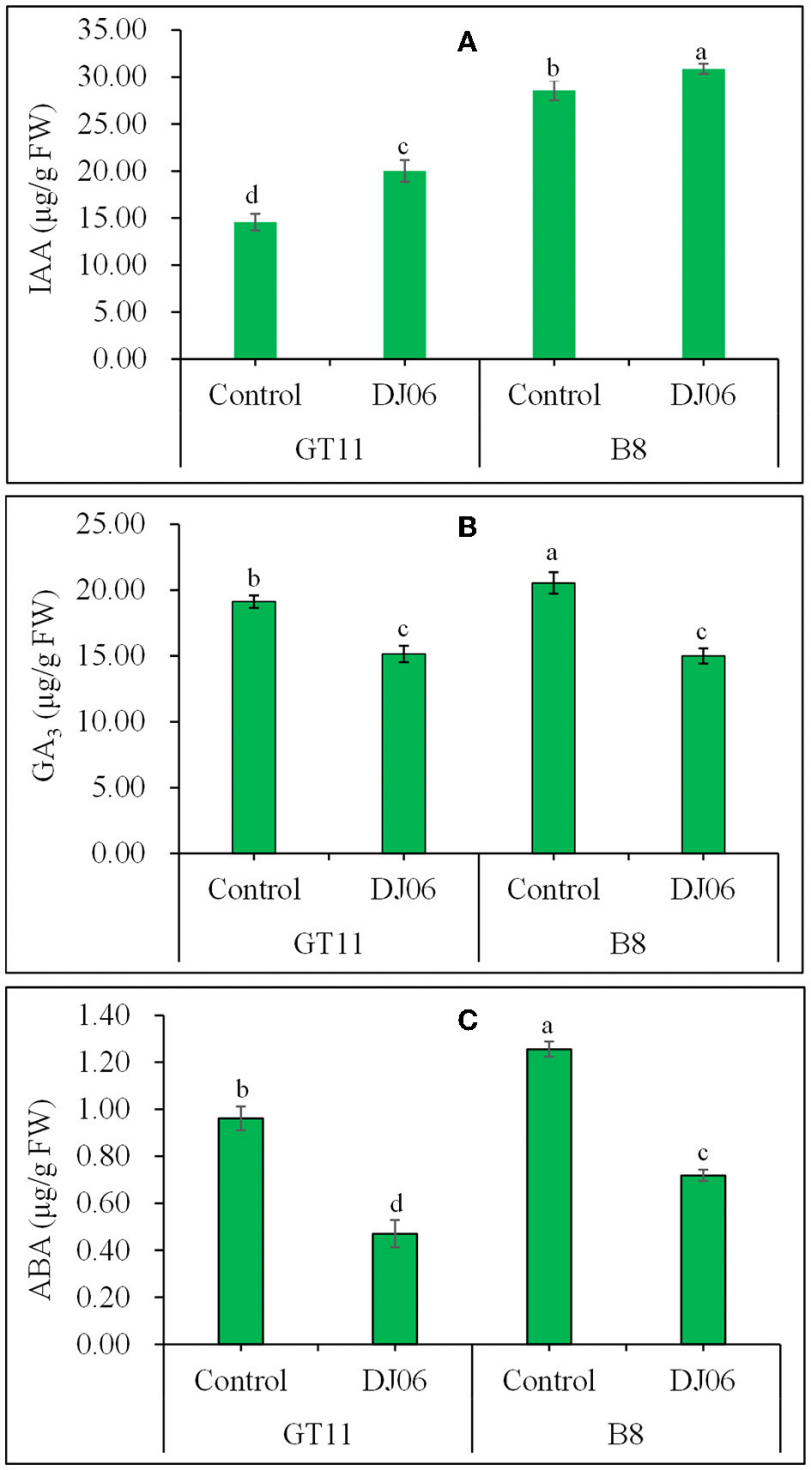
The endogenous hormones in sugarcane varieties GT11 **(A)** and B8 **(B)** 60 days after inoculation with *Pseudomonas aeruginosa* DJ06 compared with the control. **(A)** IAA; **(B)** GA_3_; **(C)** ABA. The same letter indicated that no significant difference was detected at Duncan's multiple range test, *P* ≤ 0.05 (*n* = 3).

A total of three endogenous plant hormones, namely IAA, GA_3_, and ABA, were examined. The IAA content in sugarcane varieties GT11 and B8 was increased by 37.38 and 8.01%, whereas the contents of GA_3_ and ABA in GT11 and B8 significantly decreased at 60 days after inoculation with the DJ06 strain as compared with the controls (Figure 6).

### Plant physiology relevant enzyme activities

**Figure 7:**
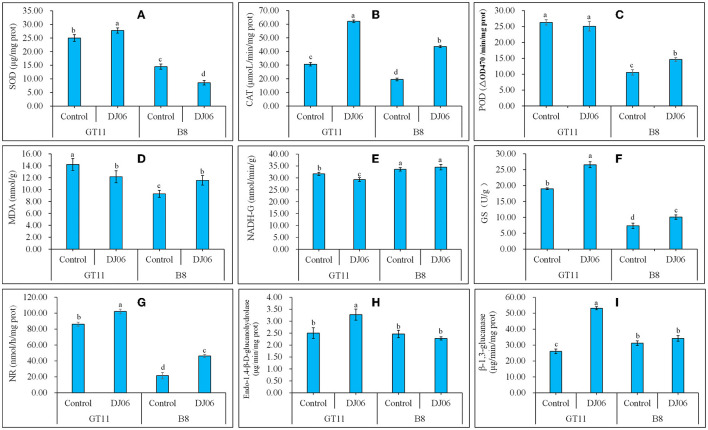
The activities of various physiological enzymes and the content of MDA in sugarcane varieties GT11 and B8 60 days after inoculation with *Pseudomonas aeruginosa* DJ06 compared with the control. **(A)** SOD; **(B)** CAT; **(C)** POD; **(D)** MDA; **(E)** NADH-GDH; **(F)** GS; **(G)** NR; **(H)** Endo-1,4-β-D-glucanohydrolase; **(I)**, β-1,3-glucanase. The same letter indicated that no significant difference was detected at Duncan's multiple range test, *P* ≤ 0.05 (*n* = 3).

The activities of four oxidative enzymes were tested, and the results showed that the SOD activity in sugarcane variety GT11 rose by 11.35% whereas it decreased by 40.79% in B8 as compared to the control. However, the CAT activity in two sugarcane varieties exhibited the same change trend and was enhanced by 102.77 and 179.08% in GT11 and B8, respectively. The POD activity in the two sugarcane varieties was inconsistent, specifically that in B8 was higher but that in GT11 showed no change when compared to the control. The change of the MDA content in B8 was similar to the POD activity, which was enhanced by 24.43% at 60 days after inoculation with the DJ06 strain. However, that in GT11 was lowered by 14.48% (Figures 7A–D).

Nitrogen metabolism-related enzyme activities were also assessed in the present study. The DADH-GDH activity in sugarcane variety GT11 decreased significantly by 7.54% at 60 days after DJ06 inoculation when compared to the control, and there was no significant difference in B8. However, the GS and NR activities significantly increased at 60 days in two sugarcane varieties, that is, GS activity was enhanced by 39.77 and 37.33%, and the NR activity was increased by 18.61 and 119.08%, respectively (Figures 7E–G). A total of two hydrolases, endo-1,4-β-D-glucanohydrolase and β-1,3-glucanase, expressed similar results. The activities of two hydrolases in GT11 significantly increased by 31.20 and 104.11% at 60 days, while those in B8 were not significantly different in the inoculation treatment as compared to the control (Figures 7H, I).

## Discussion

Sugarcane is a sugar and energy crop, and farmers apply a large amount of chemical fertilizers to increase sugarcane yields. However, the excessive application of chemical fertilizers not only enhances the cost of sugarcane production but also causes severe pollution to the soil environment and groundwater (Bokhtiar and Sakurai, 2005; Jiang et al., 2012; Li et al., 2016, 2020). Nitrogen is an essential element for plant growth, whereas the source of nitrogen for crops mainly depends on synthetic fertilizers. Biological nitrogen fixation (BNF) is an ecological approach to improve crop production and reduce the application of chemical nitrogen fertilizers (Rosenblueth et al., 2018). In this study, the *P. aeruginosa* strain DJ06 has vigorous nitrogenase activity. A series of nitrogen metabolism-related genes were found in the genome of *P. aeruginosa* DJ06, which were mainly involved in nitrogen fixation, ammonia assimilation, and nitrosative stress. Nitrogen fixation mainly involves four genes, i.e., *iscU* and three unknown genes. iscU protein is necessary for biological nitrogen fixation and plays a significant role in Fe-S cluster aggregation because the NifU nitrogen fixation protein is encoded by the *iscU* gene (Smith et al., 2005; Crooks et al., 2012). According to Andrés-Barrao et al. (2017), nitrogenase-encoding gene *nifHDK* was also found in strain *Enterobactor* sp. SA187 genome. The genomes of *Klebsiella variicola* GN02, *K. variicola* DX120E, *Enterobacter roggenkampii* ED5, and *streptomyces chartreuses* WZS021 also contain several nitrogen fixation-related genes, such as *nif* gene clusters, *nifHDK, iscU*, and *nifLA*; nitrogen metabolism regulator genes, *ntrBC* and *glnD*; and ammonia assimilation cycle gene, *amtB* (Lin et al., 2015, 2019; Wang et al., 2019; Guo et al., 2020). In this study, DNDH-GDH, GS, and NR activities were examined with the inoculated strain DJ06, and the results showed that sugarcane nitrogen metabolism enzyme activities were changed after inoculation of the DJ06 strain. The nitrate reductase activity was affected by nitrogen-fixing bacteria *Herbaspirillum seropedicae* when inoculated at two nitrogen application levels (3.0 and 0.3 mM) (da Fonseca Breda et al., 2019). Similar to this study, the inoculation of *Bacillus tequilensis* SX31 in cucumber for 3 weeks increased the activities of nitrogen metabolism-related enzymes (nitrate reductase, glutamine synthase, glutamine-2-oxoglutarate acid aminotransferase, and glutamate dehydrogenase) in cucumber (Wang et al., 2022).

Like nitrogen, phosphorus is another major element for plant growth. Phosphorous usually exists in the soil as insoluble and cannot be directly absorbed by plants. Some PGPB strains can dissolve phosphate in the soil as orthophosphate (${\text{PO}}_{4}^{3-}$) to provide growth and development for plants (Etesami, 2020). In this study, 11 endophytic strains exhibited the phosphate solubilization trait, and the DJ06 strain showed the most potent ability. Similar to our research, other *Pseudomonas* stains, such as *P. sativum* L (Oteino et al., 2015), *P. aeruginosa* KUPSB12 (Paul and Sinha, 2017), and *P. plecoglossicida* (Astriani et al., 2020), were also reported as phosphate solubilizers. The genome of the DJ06 strain contains 11 genes related to phosphorus metabolisms, such as *phoADBRU, pit, pstABCS*, and one unnamed gene. The *Pit* system is constitutive (Jansson, 1988). However, the *Pst* transporter is phosphate-inhibited and induced by phosphate-limiting conditions. Kwak et al. (2016) documented that *P. lutea* OK2T has potential PGP characteristics, including the ability to dissolve phosphate, and a large-scale value. The *PhoB* transcriptional regulator, part of the *Phog*-*PhoR* two-component signaling system, senses inorganic phosphate restriction, thereby turning on the expression of the *vreA, vreI*, and *vreR* genes that make up the operon (Faure et al., 2013).

The secretion of IAA is another direct plant-promoting characteristic of PGPBs such as *P. fluorescens* (Gravel et al., 2007), *P. aeruginosa* TQ3 (Khare and Arora, 2010), and *P. putida*1290 (Leveau and Lindow, 2005). In this study, the genome sequencing revealed that the genome of the DJ06 strain included the gene *trpCGES* encoding the enzyme related to the IAA synthesis pathway and three unknown genes for auxin efflux carrier. In addition, similar to our study, the presence of tryptophan-related genes is associated with IAA production in previously published bacterial genomes (Naveed et al., 2015; Liu et al., 2019). It was reported that *P. aeruginosa* 6A (BC4) (Marathe et al., 2017) and *P. putida* UB1 (Bharucha et al., 2013) enhance plant IAA levels and stimulate plant development. Previous research demonstrated that the tryptophan biosynthesis gene *trpABD* was involved in IAA production in the genome of *Sphingomonas* sp. LK11, and the siderophores secreted by PGPR strains are essential for plant growth under plant iron nutrient limitations (Asaf et al., 2018). In addition, the indole-3-pyruvate decarboxylase gene *ipd* was found in the genome of the strain *Acinetobacter calcoaceticus* SAVSo04, which produced high IAA *in vitro* (Leontidou et al., 2020). In the present study, the endogenous hormone IAA in the plant was significantly enhanced in two sugarcane varieties, GT11 and B8, as compared to the control at 60 days after inoculation.

Kurepin et al. (2015) reported that the root growth of potatoes correlated with the levels of phytohormones, IAA and GA_1_, after inoculation with *Burkholderia phytofirmans* PsJN. Under cadmium (Cd) stress, *Brassica nigra* L. was inoculated with high IAA-producing strains *Lysinibacillus varians* and *P. putida*, and the results showed that the plant germination rate, root and stem length, chlorophyll content, and other growth parameters were all improved (Pal et al., 2019). The contents of GA_3_ and ABA in the plant of sugarcane varieties, GT11 and B8, were decreased with the inoculation of the DJ06 strain in the present study. However, during salinity stress with inoculation of *Bacillus subtilis*, the contents of IAA, ABA, N, P, K+, Ca^2+^, and Mg^2+^ enhanced significantly in radish (*Raphanus sativus*) in relation to control plants. In contrast, the contents of ABA, Na^+^, and Cl^−^ significantly decreased, indicating that the inoculation with PGPB significantly changed the endogenous plant hormones (Mohamed and Gomaa, 2012).

The ability of the DJ06 strain to siderophore secretion was confirmed in the PGP test. Its encoding genes are mainly involved in siderophore transmembrane transporter activity, siderophore biosynthetic process, and TonB-dependent receptor, including various genes such as *tonB, fabY, fur, fepA, fecAEI*, and *fhuE*. Similar to the present study, the siderophore synthesis pathway-related gene *fepEGDC* also exists in the genome of strain *B. subtilis* EA-CB0575 (Franco-Sierra et al., 2020).

In the present study, we predicted the HCN and phenazine synthesis-related genes in the DJ06 strain genome. HCN is a volatile secondary metabolite that is naturally produced by bacteria and has an antagonistic effect against phytopathogenic fungi. It has also been suggested that HCN has a fixative effect on iron, thereby directly increasing the availability of phosphate to promote plant growth (Sagar et al., 2018). Currently, the molecular data of HCN synthase are mainly related to *Pseudomonas* strains from the GenBank database (Rijavec and Lapanje, 2017). This study found cyanide-forming glycine dehydrogenase subunit-coding gene *hcnABC* in the DJ06 strain genome. Real-time reverse transcription PCR (qRT-PCR) analysis indicated that the expression of the *hcnC* gene in *Pseudomonas sp*. LBUM300 inoculated to strawberries was significantly stimulated by the infection of *Verticillium dahlia*, and the number of *Pseudomonas* strains was increased (DeCoste et al., 2010). Phenazine is another class of nitrogen-containing pigment secondary metabolite secreted by *Pseudomonas* with a significant ability to inhibit phytopathogens. Approximately 100 different phenazine derivatives have been identified recently, and 6,000 related compounds have been synthesized (Bilal et al., 2017). A total of two redundant operons, *phzA1-G1* (*phz1*) and *phzA2-G2* (*phz2*), in *P. aeruginosa* have been reported to encode nearly identical proteins, which are precursors to several phenazine derivatives (Recinos et al., 2012). In this study, the phenazine synthesis-coding genes, *phzA_B* and *phzF*, were found in the genome of the DJ06 strain. Similarly, these genes were also predicted in *P*. *chlororaphis* GP72 (Shen et al., 2013), *Pantoea agglomerans* C1 (Luziatelli et al., 2020), and *P. aeruginosa* B18 (Singh et al., 2021). In addition, we observed several gene clusters related to hydrolase synthesis in the DJ06 strain genome, including *pslG, bglBX, folE2, folE, ribA, ribBA*, and some unknown genes. These genes encode the proteins of chitinase, cellulase, β-glucosidase, etc., which effectively destroy the cell wall of phytopathogenic fungi to realize biocontrol. Similar genes have also been predicted in other strain genomes (Shariati et al., 2017; Luo et al., 2018).

Abiotic stress can cause severe damage to plant productivity and sometimes total mortality (Mittler, 2006). Currently, a variety of PGPB strains have been successfully applied to alleviate crop abiotic stresses. In this study, we predicted a large number of drought resistance and heavy metal toxicity-related genes in the genome of the DJ06 strain related to magnesium (Mg) transport (*corAC*), copper (Cu) homeostasis (*copABZ, cusSR*), zinc (Zn) homeostasis (*znuABC*), chromium homeostasis (*copB, czcACD*), etc. These genes encode the proteins associated with the tolerance to divalent cations of Mg, Cu, Zn, and chromium due to their ability to automatically efflux metal ions (Mima et al., 2009). Cu is an essential component of biological evolution and a metal cofactor for several enzymes such as monooxygenase, dioxygenase, and SOD (Giner-Lamia et al., 2012). Excess Cu harms plants by inhibiting shoot and root growth, reducing respiration and photosynthesis, and altering enzymatic activities (Rajput et al., 2018). Zn is transferred in the form of Zn^2+^ and absorbed by plants for maintaining biological structural and functional integrity, promoting protein synthesis, gene expression, enzyme structure, energy production, and positively impacting crop plants, especially in plant productivity (Mousavi et al., 2013). However, excess Zn may inhibit the activity of plant photosystem II (PS II), decrease RUBP carboxylase, and induce Zn toxicity when the concentration exceeds 300 ppm (Prasad et al., 1999). The ability to resist Zn is different in diverse plants (Vitosh et al., 1994). Therefore, various metal ion metabolism-related genes in the DJ06 genome may play an essential role in mitigating heavy metal toxicity. In addition, the transpiration rates in the two sugarcane varieties, GT11 and B8, showed differences after inoculation with the DJ06 strain. We speculate that it may be due to the genetic differences between sugarcane varieties.

Some PGPB strains could alleviate drought stress in the plant growth process. It was reported that PGPB strains secreted 1-aminocyclopropane-1-carboxylate (ACC) deaminase (EC 4.1.99.4), which catalyzes ACC to α-ketobutyric acid when plants respond to biotic/abiotic stresses and ammonia to regulate ethylene stress to protect plants from damage (Danish et al., 2020; Ullah et al., 2021). The ACC deaminase-encoding gene was also found in the DJ06 genome, and similar genes also appeared in the genomes of strains ED5 and B18 (Guo et al., 2020; Singh et al., 2021). Currently, the PGPB strains, which produce ACC deaminase, are widely used in the study of drought resistance of crops, such as *B. amyloliquefaciens* (Danish and Zafar-Ul-Hye, 2019), *Agrobacterium fabrum* (Munir and Zafarul-Hye, 2019), and *Pseudomonas* spp. (Shaharoona and Mahmood, 2008). In addition, drought stress-related genes, such as *nhaB, kdpABCDE, GDT1*, and *proCX*, were also predicted in the DJ06 genome. At the same time, it was found that the activities of oxidative stress enzymes (SOD, POD, and CAT) and the content of oxidative product MDA in sugarcane varieties, GT11 and B8, inoculated with the DJ06 strain was significantly changed as compared with the controls in this study, indicating that the DJ06 strain stimulated the occurrence of physiological responses of sugarcane, and then enhance the ability to adapt to the external environment. The variations in SOD, POD, and MDA activities after inoculation of the DJ06 strain may differ from the genetic variability in the two sugarcane varieties (Guo et al., 2021). In addition, this study found that there were mobile genetic elements (MGEs) in the genome of the DJ06 strain, such as the CRISPR system and prophage, which play an important role in PGPB strains for adaptation to the environment, and similar results were also reported in *Enterobacter roggenkampii* ED5 (Guo et al., 2020).

Sulfur is a vital nutrient element for plant growth and development, which is related to plant stress resistance (Gill and Tuteja, 2011), and sulfur deficiency in crops will lead to severe yield losses. Some scholars have found the function of the *cysP* gene in *Bacillus subtilis* by transforming *Escherichia coli* with the plasmid of the *cysP* gene through the sulfate transport mutant; that is, the *cysP* gene operon in *B. subtilis* is responsible for sulfur metabolism (Aguilar-Barajas et al., 2011). Some transporter-related genes, such as *cysABCDEGKHIUWSPZ*, were found in the DJ06 genome. It has been reported that these genes may be involved in the transport of thiosulfate or inorganic sulfate into cells (Duan et al., 2013) and possibly the oxidation of sulfur and sulfur-conjugated secondary metabolites (Kwak et al., 2014). In addition, sulfur oxidation affects soil pH and gradually increases the solubility of nutrients, such as N, P, K, Mg, and Zn, which can enhance plant uptake of mineral nutrients (Wainwright, 1984).

Plant growth-promoting bacteria (PGPB) usually colonize inside different parts of plants, such as roots, stems, and leaves, and perform beneficial functions (Liu et al., 2011). The colonization of PGPB in plants is a prerequisite for mutualistic symbiosis. In this study, the SEM and GFP labeling techniques were used to confirm that the DJ06 strain colonized in sugarcane tissues successfully. The analysis of *Pseudomonas aeruginosa* DJ06 revealed that many colonization-related and signal transduction chemotaxis genes, such as *cheABDRVWYZ, pilIJK, aer*, and *mcp*, play essential roles in microbe–plant interactions (Drr et al., 2010). Similar results were also found in the genome of the strain *P. fluorescens* PCL1751 (Cao et al., 2015).

Biofilms are constrained by polymers, such as autogenic exopolysaccharides, extracellular DNA, and biological surface-associated proteins (Bogino et al., 2013; Teschler et al., 2015). PGPBs in plants' interior or rhizosphere region form biofilms to coexist with plants. PGPBs mutually beneficially coexist with plants by forming biofilms inside plant roots or in the rhizosphere region. Previous reports showed the antagonistic activities of *Paeni bacillus, B. cereus*, and *P. stutzeri* against phytopathogens by initiating biofilms (Xu et al., 2014; Salme et al., 2015; Wang et al., 2017). In biofilms, cell-to-cell communication enhances gene upregulation and downregulation, thereby improving the fitness of microorganisms in both biotic and abiotic environments. In the genome of the DJ06 strain, several flagellar motility protein-encoding genes related to biogenesis were also found, such as *flgABCDEFGHIJKLMN* and *motAB*, which indicated that the DJ06 strain has potential plant growth-promoting properties.

## Conclusion

This study showed the plant growth-promoting effects of endophytic PGP strain *P. aeruginosa* DJ06 isolated from sugarcane root. It was confirmed that the DJ06 strain could successfully colonize sugarcane tissue. The complete genome study of the DJ06 strain exhibited the presence of various PGP genes in its genome. The inoculation of the DJ06 strain inoculation also significantly increased some agronomic parameters, i.e., plant height, fresh weight, and photosynthesis in sugarcane varieties GT11 and B8 as compared with the control under greenhouse conditions. In addition, the endogenous plant hormones and physiological enzyme activities in the two sugarcane varieties also changed significantly after the inoculation. Our findings provide a reference for the molecular interaction mechanism between the DJ06 strain and sugarcane.

## Data availability statement

The datasets presented in this study can be found in the NCBI repository, accession number CP080511: https://www.ncbi.nlm.nih.gov/nuccore/CP080511.

## Author contributions

D-JG, D-PL, RS, and Y-RL: planned the proposal and experiments. D-JG and D-PL: accomplished the experiments. KV, QK, YQ, T-SC, B-QZ, and X-PS: data examination. Y-RL: study and resources. D-JG and PS: writing the original manuscript. BY and Y-RL: review and editing. All authors contributed to the article and approved the submitted version.

## References

[B1] Aguilar-Barajas E. Díaz-Pérez C. Ramírez-Díaz M. Riveros-Rosas H. Cervantes C. (2011). Bacterial transport of sulfate, molybdate, and related oxyanions. BioMetals. 24, 687–707. 10.1007/s10534-011-9421-x 21301930

[B2] Andrés-Barrao C. Lafi F. F. Alam I. De Zélicourt A. Eida A. A. Bokhari A.. (2017). Complete genome sequence analysis of *Enterobacter* sp. SA187, a plant multi-stress tolerance promoting endophytic bacterium. Front. Microbiol. 8, 2023. 10.3389/fmicb.2017.02023 29163376 PMC5664417

[B3] Antunes J. E. L. Freitas A. D. S. Oliveira L. Lyra M. DO Carmo C. D. Fonseca M. A.. (2019). Sugarcane inoculated with endophytic diazotrophic bacteria: effects on yield, biological nitrogen fixation and industrial characteristics. An. Acad. Bras. Cienc. 91, 2019. 10.1590/0001-3765201920180990 31778453

[B4] Asaf S. Khan A. L. Khan M. A. Al-Harrasi A. Lee I.-J. (2018). Complete genome sequencing and analysis of endophytic *Sphingomonas* sp. LK11 and its potential in plant growth. 3 Biotech. 8, 1–14. 10.1007/s13205-018-1403-z 30175026 PMC6111035

[B5] Astriani M. Zubaidah S. Abadi A. L. Suarsini E. (2020). *Pseudomonas plecoglossicida* as a novel bacterium for phosphate solubilizing and indole-3-acetic acid-producing from soybean rhizospheric soils of east java, indonesia. Biodiversitas J. Bio. Diversity. 21, 578–586. 10.13057/biodiv/d210220

[B6] Bharathalakshmi M. Jamuna P. (2019). *Bacillus amyloliquefaciens* (RB19): A potential PGPR in managing sugarcane red rot disease. J. Pharm. Phytochem. 8, 2255–2261.

[B7] Bhardwaj G. Shah R. Joshi B. Patel P. (2017). *Klebsiella pneumoniae* VRE36 as a PGPR isolated from *Saccharum officinarum* cultivar Co99004. J. Appl. Biol. Biotechnol. 5, 47–52. 10.7324/JABB.2017.50108

[B8] Bharucha U. Patel K. Trivedi U. B. (2013). Optimization of indole acetic acid production by *Pseudomonas putida* UB1 and its effect as plant growth-promoting rhizobacteria on mustard (Brassica nigra). Agr. Res. 2, 215–221. 10.1007/s40003-013-0065-7

[B9] Bilal M. Guo S. Iqbal H. Hu H. Wang W. Zhang X. (2017). Engineering *Pseudomonas* for phenazine biosynthesis, regulation, and biotechnological applications: a review. World J. Microb. Biot. 33, 1–11. 10.1007/s11274-017-2356-9 28975557

[B10] Bogino P. Oliva M. Sorroche F. Giordano W. (2013). The role of bacterial biofilms and surface components in plant-bacterial associations. Int. J. Mol. Sci. 14, 15838. 10.3390/ijms140815838 23903045 PMC3759889

[B11] Bokhtiar S. Sakurai K. (2005). Effects of organic manure and chemical fertilizer on soil fertility and productivity of plant and ratoon crops of sugarcane. Arch. Agron. Soil Sci. 51, 325–334. 10.1080/03650340500098006

[B12] Cao S. T. C Chang H. H. Egamberdieva D. Kamilova F. Lugtenberg B.. (2015). Genome Analysis of *Pseudomonas fluorescens* PCL1751: A rhizobacterium that controls root diseases and alleviates salt stress for its plant host. PLoS ONE. 10, e0140231. 10.1371/journal.pone.0140231 26452056 PMC4599888

[B13] Ciufo S. Kannan S. Sharma S. Badretdin A. Clark K. Turner S.. (2018). Using average nucleotide identity to improve taxonomic assignments in prokaryotic genomes at the NCBI. Int. J. Syst. Evol. Micr. 68, 2386. 10.1099/ijsem.0.002809 29792589 PMC6978984

[B14] Crooks D. R. Jeong S. Y. Tong W.-H. Ghosh M. C. Olivierre H. Haller R. G.. (2012). Tissue specificity of a human mitochondrial disease: differentiation-enhanced mis-splicing of the Fe-S scaffold gene ISCU renders patient cells more sensitive to oxidative stress in ISCU myopathy. J. Biol. Chem. 287, 40119–40130. 10.1074/jbc.M112.418889 23035118 PMC3504726

[B15] da Fonseca Breda F. A. da Silva T. F. R. Dos Santos S. G. Alves G. C. Reis V. M. (2019). Modulation of nitrogen metabolism of maize plants inoculated with *Azospirillum brasilens*e and *Herbaspirillum seropedicae*. Arch. Microbiol. 201, 547–558. 10.1007/s00203-018-1594-z 30448870

[B16] Danish S. Zafar-Ul-Hye M. (2019). Co-application of ACC-deaminase producing PGPR and timber-waste biochar improves pigments formation, growth and yield of wheat under drought stress. Sci. Rep. 9, 1–13. 10.1038/s41598-019-42374-9 30979925 PMC6461675

[B17] Danish S. Zafar-Ul-Hye M. Hussain S. Riaz M. Qayyum M. F. (2020). Mitigation of drought stress in maize through inoculation with drought tolerant ACC deaminase containing PGPR under axenic conditions. Pak. J. Bot. 52, 49–60. 10.30848/PJB2020-1(7)

[B18] DeCoste N. J. Gadkar V. J. Filion M. (2010). Verticillium dahliae alters *Pseudomonas* spp. populations and HCN gene expression in the rhizosphere of strawberry. Can. J. Microbiol. 56, 906–915. 10.1139/W10-080 21076481

[B19] Drr J. Hurek T. Reinhold-Hurek B. (2010). Type IV pili are involved in plant-microbe and fungus-microbe interactions. Mol. Microbiol. 30, 7–17. 10.1046/j.1365-2958.1998.01010.x 9786181

[B20] Duan J. Wei J. Cheng Z. Heikkila J. J. Glick B. R. John V. (2013). The complete genome sequence of the plant growth-promoting *bacterium Pseudomonas* sp. UW4. PLoS ONE. 8, e58640. 10.1371/journal.pone.0058640 23516524 PMC3596284

[B21] Edwards U. Rogall T. Blöcker H. Emde M. Böttger E. C. (1989). Isolation and direct complete nucleotide determination of entire genes. Characterization of a gene coding for 16S ribosomal RNA. Nucleic Acids Res. 17, 7843–7853. 10.1093/nar/17.19.7843 2798131 PMC334891

[B22] Etesami H. (2020). “Enhanced phosphorus fertilizer use efficiency with microorganisms,” in Nutrient Dynamics for Sustainable Crop Production (Springer) 215–245. 10.1007/978-981-13-8660-2_8

[B23] Faure L. M. Llamas M. A. Bastiaansen K. C. de Bentzmann S. Bigot S. (2013). Phosphate starvation relayed by PhoB activates the expression of the *Pseudomonas aeruginosa* σvreI ECF factor and its target genes. Microbiol. 159, 1315–1327. 10.1099/mic.0.067645-0 23657684

[B24] Feng Y. Huang Y. Zhan H. Bhatt P. Chen S. (2020). An overview of strobilurin fungicide degradation: current status and future perspective. Front. Microbiol. 11, 389. 10.3389/fmicb.2020.00389 32226423 PMC7081128

[B25] Franco-Sierra N. D. Posada L. F. Santa-María G. Romero-Tabarez M. Villegas-Escobar V. Álvarez J. C. (2020). *Bacillus subtilis* EA-CB0575 genome reveals clues for plant growth promotion and potential for sustainable agriculture. Funct. Integr. Genomic. 20, 575–589. 10.1007/s10142-020-00736-x 32198678

[B26] Fukami J. Cerezini P. Hungria M. (2018). Azospirillum: benefits that go far beyond biological nitrogen fixation. Amb Express. 8, 1–12. 10.1186/s13568-018-0608-1 29728787 PMC5935603

[B27] García J. E. Maroniche G. Creus C. Suárez-Rodríguez R. Ramirez-Trujillo J. A. Groppa M. D. (2017). In vitro PGPR properties and osmotic tolerance of different *Azospirillum* native strains and their effects on growth of maize under drought stress. Microbiol. Res. 202, 21–29. 10.1016/j.micres.2017.04.007 28647119

[B28] Gill S. S. Tuteja N. (2011). Cadmium stress tolerance in crop plants: probing the role of sulfur. Plant Signal. Behav. 6, 215–222. 10.4161/psb.6.2.14880 21330784 PMC3121981

[B29] Giner-Lamia J. López-Maury L. Reyes J. C. Florencio F. J. (2012). The CopRS two-component system is responsible for resistance to copper in the cyanobacterium *Synechocystis* sp. PCC 6803. Plant Physiol. 159, 1806–1818. 10.1104/pp.112.200659 22715108 PMC3425214

[B30] Gravel V. Antoun H. Tweddell R. (2007). Growth stimulation and fruit yield improvement of greenhouse tomato plants by inoculation with *Pseudomonas putida* or *Trichoderma atroviride*: possible role of indole acetic acid (IAA). Soil Biol. Biochem. 39, 1968–1977. 10.1016/j.soilbio.2007.02.015

[B31] Gu G. Gonzalez-Escalona N. Zheng J. Bolten S. Luo Y. Mafiz A. I.. (2020). Genome sequences of *Brevundimonas naejangsanensis* strain FS1091 and *Bacillus amyloliquefaciens* strain FS1092, isolated from a fresh-cut-produce-processing plant. Microbiol. Resour. Announce. 9, e01448–e01419. 10.1128/MRA.01448-19 31974158 PMC6979307

[B32] Guo D.-J. Li D.-P. Singh R. K. Singh P. Sharma A. Verma K. K.. (2021). Differential protein expression analysis of two sugarcane varieties in response to diazotrophic plant growth-promoting endophyte *Enterobacter roggenkampii* ED5. Front. Plant Sci. 12, 2567. 10.3389/fpls.2021.727741 34887881 PMC8649694

[B33] Guo D.-J. Singh R. K. Singh P. Li D.-P. Sharma A. Xing Y.-X.. (2020). Complete genome sequence of *Enterobacter roggenkampii* ED5, a nitrogen fixing plant growth promoting endophytic bacterium with biocontrol and stress tolerance properties, isolated from sugarcane root. Front. Microbiol. 11, 580081. 10.3389/fmicb.2020.580081 33072048 PMC7536287

[B34] Hardy R. W. F. Holsten R. D. Jackson E. K. Burns R. C. (1968). The acetylene ethylene assay for N_2_ fixation: laboratory and field evaluation. Plant Physiol. 43, 1185–1207. 10.1104/pp.43.8.1185 16656902 PMC1086994

[B35] Jacobson C. B. Pasternak J. J. Glick B. R. (1994). Partial purification and characterization of 1-aminocyclopropane-1-carboxylate deaminase from the plant growth promoting rhizobacterium *Pseudomonas putida* GR12-2. Can. J. Microbiol. 40, 1019–1025. 10.1139/m94-162

[B36] Jansson M. (1988). Phosphate uptake and utilization by bacteria and algae. Hydrobiologia. 170, 177–189. 10.1007/BF00024904

[B37] Jiang Z.-P. Li Y.-R. Wei G.-P. Liao Q. Su T.-M. Meng Y.-C.. (2012). Effect of long-term vinasse application on physico-chemical properties of sugarcane field soils. Sugar Tech. 14, 412–417. 10.1007/s12355-012-0174-9

[B38] Khare E. Arora N. K. (2010). Effect of indole-3-acetic acid (IAA) produced by *Pseudomonas aeruginosa* in suppression of charcoal rot disease of chickpea. Curr. Microbiol. 61, 64–68. 10.1007/s00284-009-9577-6 20049597

[B39] Kloepper J. W. Leong J. Teintze M. Schroth M. N. (1980). Enhanced plant growth by siderophores produced by plant growth-promoting rhizobacteria. Nature. 286, 885–886. 10.1038/286885a0

[B40] Kurepin L. V. Park J. M. Lazarovits G. Bernards M. A. (2015). *Burkholderia phytofirmans-*induced shoot and root growth promotion is associated with endogenous changes in plant growth hormone levels. Plant Growth Regul. 75, 199–207. 10.1007/s10725-014-9944-6

[B41] Kwak M. J. Jeong H. Madhaiyan M. Lee Y. Sa T. M. Oh T. K.. (2014). Genome information of *Methylobacterium oryzae*, a plant-probiotic methylotroph in the phyllosphere. PLoS ONE. 9, 704. 10.1371/journal.pone.0106704 25211235 PMC4161386

[B42] Kwak Y. Park G.-S. Shin J.-H. (2016). High quality draft genome sequence of the type strain of *Pseudomonas lutea* OK2T, a phosphate-solubilizing rhizospheric bacterium. Stand. Genomic Sci. 11, 1–10. 10.1186/s40793-016-0173-7 27555890 PMC4994261

[B43] Lamizadeh E. Enayatizamir N. Motamedi H. (2016). Isolation and identification of plant growth-promoting rhizobacteria (PGPR) from the rhizosphere of sugarcane in saline and non-saline soil. Int. J. Curr. Microbiol. Appl. Sci. 5, 1072–1083. 10.20546/ijcmas.2016.510.113

[B44] Leontidou K. Genitsaris S. Papadopoulou A. Kamou N. Bosmali I. Matsi T.. (2020). Plant growth promoting rhizobacteria isolated from halophytes and drought-tolerant plants: Genomic characterisation and exploration of phyto-beneficial traits. Sci. Rep. 10, 1–15. 10.1038/s41598-020-71652-0 32908201 PMC7481233

[B45] Leveau J. H. Lindow S. E. (2005). Utilization of the plant hormone indole-3-acetic acid for growth by *Pseudomonas putida* strain 1290. Appl. Environ. Microb. 71, 2365–2371. 10.1128/AEM.71.5.2365-2371.2005 15870323 PMC1087548

[B46] Li H.-B. Singh R. K. Singh P. Song Q.-Q. Xing Y.-X. Yang L.-T.. (2017). Genetic diversity of nitrogen-fixing and plant growth promoting *Pseudomonas* species isolated from sugarcane rhizosphere. Front. Microbiol. 8, 1268. 10.3389/fmicb.2017.01268 28769881 PMC5509769

[B47] Li Y. Are K. S. Huang Z. Guo H. Wei L. Abegunrin T. P.. (2020). Particulate N and P exports from sugarcane growing watershed are more influenced by surface runoff than fertilization. Agr. Ecosyst. Environ. 302, 107087. 10.1016/j.agee.2020.107087

[B48] Li Y.-R. Song X.-P. Wu J.-M. Li C.-N. Liang Q. Liu X.-H.. (2016). Sugar industry and improved sugarcane farming technologies in China. Sugar Tech. 18, 603–611. 10.1007/s12355-016-0480-8

[B49] Li Y.-R. Yang L.-T. (2015). Sugarcane agriculture and sugar industry in China. Sugar Tech 17, 1–8. 10.1007/s12355-014-0342-1

[B50] Li Z. Chang S. Lin L. Li Y. An Q. (2011). A colorimetric assay of 1-aminocyclopropane-1-carboxylate (ACC) based on ninhydrin reaction for rapid screening of bacteria containing ACC deaminase. Lett. Appl. Microbiol. 53, 178–185. 10.1111/j.1472-765X.2011.03088.x 21599721

[B51] Lin B. Song Z. Jia Y. Zhang Y. Wang L. Fan J.. (2019). Biological characteristics and genome-wide sequence analysis of endophytic nitrogen-fixing bacteria *Klebsiella variicola* GN02. Biotechnol. Biotec. Eq. 33, 108–117. 10.1080/13102818.2018.1555010

[B52] Lin L. Guo W. Xing Y. Zhang X. Li Z. Hu C.. (2012). The actinobacterium *Microbacterium* sp. 16SH accepts pBBR1-based pPROBE vectors, forms biofilms, invades roots, and fixes N_2_ associated with micropropagated sugarcane plants. *Appl. Microbiol*. Biot. 93, 1185–1195. 10.1007/s00253-011-3618-3 22002067

[B53] Lin L. Wei C. Chen M. Wang H. Li Y. Li Y.. (2015). Complete genome sequence of endophytic nitrogen-fixing *Klebsiella variicola* strain DX120E. Stand. Genomic Sci. 10, 1–7. 10.1186/s40793-015-0004-2 26203334 PMC4511632

[B54] Liu W.-H. Chen F.-F. Wang C.-E. Fu H.-H. Fang X.-Q. Ye J.-R.. (2019). Indole-3-acetic acid in *Burkholderia pyrrocinia* JK-SH007: Enzymatic identification of the indole-3-acetamide synthesis pathway. Front. Microbiol. 10, 2559. 10.3389/fmicb.2019.02559 31749788 PMC6848275

[B55] Liu Y. Wang H. Sun X. Yang H. Wang Y. Song W. (2011). Study on mechanisms of colonization of nitrogen-fixing PGPB, *Klebsiella pneumoniae* NG14 on the root surface of rice and the formation of biofilm. Curr. Microbiol. 62, 1113–1122. 10.1007/s00284-010-9835-7 21132569

[B56] Lorck H. (1948). Production of hydrocyanic acid by bacteria. Physiol. Plant. 1, 142–146. 10.1111/j.1399-3054.1948.tb07118.x

[B57] Luo Y. Cheng Y. Yi J. Zhang Z. Luo Q. Zhang D.. (2018). Complete genome sequence of industrial biocontrol strain *Paenibacillus polymyxa* HY96-2 and further analysis of its biocontrol mechanism. Front. Microbiol. 9, 1520. 10.3389/fmicb.2018.01520 30050512 PMC6052121

[B58] Luziatelli F. Ficca A. G. Cardarelli M. Melini F. Cavalieri A. Ruzzi M. (2020). Genome sequencing of *Pantoea agglomerans* C1 provides insights into molecular and genetic mechanisms of plant growth-promotion and tolerance to heavy metals. Microorg. 8, 153. 10.3390/microorganisms8020153 31979031 PMC7074716

[B59] Marathe R. Phatake Y. Shaikh A. Shinde B. Gajbhiye M. (2017). Effect of IAA produced by *Pseudomonas aeruginosa* 6a (bc4) on seed germination and plant growth of Glycin max. J. Exp. Bio.l Agric. Sci. 5, 351–358. 10.18006/2017.5(3).351.358

[B60] Meftaul I. M. Venkateswarlu K. Dharmarajan R. Annamalai P. Asaduzzaman M. Parven A.. (2020). Controversies over human health and ecological impacts of glyphosate: Is it to be banned in modern agriculture? Environ. Pollut. 263, 114372. 10.1016/j.envpol.2020.114372 32203845

[B61] Mima T. Kohira N. Li Y. Sekiya H. Ogawa W. Kuroda T.. (2009). Gene cloning and characteristics of the RND-type multidrug efflux pump MuxABC-OpmB possessing two RND components in *Pseudomonas aeruginosa*. Microbiol. 155, 3509–3517. 10.1099/mic.0.031260-0 19713238

[B62] Mirza M. S. Ahmad W. Latif F. Haurat J. Bally R. Normand P.. (2001). Isolation, partial characterization, and the effect of plant growth-promoting bacteria (PGPB) on micro-propagated sugarcane in vitro. Plant Soil. 237, 47–54. 10.1023/A:1013388619231

[B63] Mittler R. (2006). Abiotic stress, the field environment and stress combination. Trends Plant Sci. 11, 15–19. 10.1016/j.tplants.2005.11.002 16359910

[B64] Mohamed H. Gomaa E. (2012). Effect of plant growth promoting Bacillus subtilis and *Pseudomonas fluorescens* on growth and pigment composition of radish plants (Raphanus sativus) under NaCl stress. Photosynthetica. 50, 263–272. 10.1007/s11099-012-0032-8

[B65] Mousavi S. R. Galavi M. Rezaei M. (2013). Zinc (Zn) importance for crop production—a review. Int. J. Agron. Plant Prod. 4, 64–68.

[B66] Munir T. Zafarul-Hye M. (2019). ACC Deaminase Producing PGPR *Bacillus amyloliquefaciens* and *Agrobacterium fabrum* along with biochar improve wheat productivity under drought stress. Agron. 9, 1–16. 10.3390/agronomy9070343

[B67] Naveed M. Qureshi M. A. Zahir Z. A. Hussain M. B. Sessitsch A. Mitter B. (2015). L-Tryptophan-dependent biosynthesis of indole-3-acetic acid (IAA) improves plant growth and colonization of maize by *Burkholderia phytofirmans* PsJN. Ann. Microbiol. 65, 1381–1389. 10.1007/s13213-014-0976-y

[B68] Nehra V. Choudhary M. (2015). A review on plant growth promoting rhizobacteria acting as bioinoculants and their biological approach towards the production of sustainable agriculture. J. Appl. Nat. Sci. 7, 540–556. 10.31018/jans.v7i1.642

[B69] Oliveira M. Ramos E. Drechsel M. Vidal M. Schwab S. Baldani J. (2018). Gluconacin from *Gluconacetobacter diazotrophicus* PAL5 is an active bacteriocin against phytopathogenic and beneficial sugarcane bacteria. J. Appl. Microbiol. 125, 1812–1826. 10.1111/jam.14074 30136440

[B70] Oteino N. Lally R. D. Kiwanuka S. Lloyd A. Ryan D. Germaine K. J.. (2015). Plant growth promotion induced by phosphate solubilizing endophytic *Pseudomonas* isolates. Front. Microbiol. 6, 745. 10.3389/fmicb.2015.00745 26257721 PMC4510416

[B71] Pal A. K. Mandal S. Sengupta C. (2019). Exploitation of IAA producing PGPR on mustard (*Brassica nigra* L.) seedling growth under cadmium stress condition in comparison with exogenous IAA application. Plant Sci. Today. 6, 22–30. 10.14719/pst.2019.6.1.440

[B72] Paul D. Sinha S. N. (2017). Isolation and characterization of phosphate solubilizing bacterium *Pseudomonas aeruginosa* KUPSB12 with antibacterial potential from river Ganga, India. An. Agr. Sci. 15, 130–136. 10.1016/j.aasci.2016.10.001

[B73] Pikovskaya R. I. (1948). Mobilization of phosphorus in soil in connection with vital activity of some microbial species. Mikrobiologiya. 17, 362–370.

[B74] Pitiwittayakul N. Wongsorn D. Tanasupawat S. (2021). Characterisation of plant growth-promoting endophytic bacteria from sugarcane and their antagonistic activity against *Fusarium moniliforme*. Tropical Life Sci. Res. 32, 97. 10.21315/tlsr2021.32.3.6 35656370 PMC9132556

[B75] Prasad K. V. S. K. Saradhi P. P. Sharmila P. (1999). Concerted action of antioxidant enzymes and curtailed growth under zinc toxicity in *Brassica juncea*. Environ. Exp. Bot. 42, 1–10 10.1016/S0098-8472(99)00013-1

[B76] Rajput V. Minkina T. Suskova S. Mandzhieva S. Tsitsuashvili V. Chapligin V.. (2018). Effects of copper nanoparticles (CuO NPs) on crop plants: a mini review. Bionanosci. 8, 36–42. 10.1007/s12668-017-0466-3

[B77] Recinos D. A. Sekedat M. D. Hernandez A. Cohen T. S. Sakhtah H. Prince A. S.. (2012). Redundant phenazine operons in *Pseudomonas aeruginosa* exhibit environment-dependent expression and differential roles in pathogenicity. P. Natl. Acad. Sci. USA. 109, 19420–19425. 10.1073/pnas.1213901109 23129634 PMC3511076

[B78] Rijavec T. Lapanje A. (2017). Cyanogenic *Pseudomonas* spp. strains are concentrated in the rhizosphere of alpine pioneer plants. Microbiol. Res. 194, 20–28. 10.1016/j.micres.2016.09.001 27938859

[B79] Rosa P. A. L. Galindo F. S. Oliveira C. E. d. S Jalal A. Mortinho E. S.. (2022). Inoculation with plant growth-promoting bacteria to reduce phosphate fertilization requirement and enhance technological quality and yield of sugarcane. Microorg. 10, 192. 10.3390/microorganisms10010192 35056643 PMC8781176

[B80] Rosenblueth M. Ormeño-Orrillo E. López-López A. Rogel M. A. Reyes-Hernández B. J. Martínez-Romero J. C.. (2018). Nitrogen fixation in cereals. Front. Microbiol. 9, 1794. 10.3389/fmicb.2018.01794 30140262 PMC6095057

[B81] Sagar A. Dhusiya K. Shukla P. Singh A. Lawrence R. Ramteke P. (2018). Comparative analysis of production of hydrogen cyanide (HCN) with production of siderophore (SD) and phosphate solubilization (PS) activity in plant growth promoting bacteria (PGPB). Vegetos. 31, 130–135. 10.5958/2229-4473.2018.00064.2

[B82] Salme T. Seong-Bin K. Eviatar N. Islam A. E. D. Bo E. Jonas B.. (2015). Sfp-type PPTase inactivation promotes bacterial biofilm formation and ability to enhance wheat drought tolerance. Front. Microbiol. 6, 387. 10.3389/fmicb.2015.00387 26052312 PMC4439574

[B83] Savci S. (2012). Investigation of effect of chemical fertilizers on environment. APCBEE Procedia. 1, 287–292. 10.1016/j.apcbee.2012.03.047

[B84] Schwyn B. Neilands J. B. (1987). Universal chemical assay for the detection and determination of siderophores. Anal. Biochem. 160, 47–56. 10.1016/0003-2697(87)90612-9 2952030

[B85] Shaharoona B. Mahmood T. (2008). Inoculation with *Pseudomonas spp*. containing acc-deaminase partially eliminates the effects of drought stress on growth, yield, and ripening of pea (*Pisum sativum L*.). Pedosphere. 18, 611–620. 10.1016/S1002-0160(08)60055-7

[B86] Shariati J. V Malboobi M. A. Tabrizi Z. Tavakol E. Owlia P.. (2017). Comprehensive genomic analysis of a plant growth-promoting rhizobacterium *Pantoea agglomerans* strain P5. Sci. Rep. 7, 1–12. 10.1038/s41598-017-15820-9 29142289 PMC5688152

[B87] Sharma A. Singh R. K. Singh P. Vaishnav A. Guo D.-J. Verma K. K.. (2021). Insights into the bacterial and nitric oxide-induced salt tolerance in sugarcane and their growth-promoting abilities. Microorg. 9, 2203. 10.3390/microorganisms9112203 34835329 PMC8623439

[B88] Shen X. Hu H. Peng H. Wang W. Zhang X. (2013). Comparative genomic analysis of four representative plant growth-promoting rhizobacteria in *Pseudomonas. BMC Genom*.14, 1–20. 10.1186/1471-2164-14-271 23607266 PMC3644233

[B89] Singh P. Singh R. K. Guo D.-J. Sharma A. Singh R. N. Li D.-P.. (2021). Whole genome analysis of sugarcane root-associated endophyte *Pseudomonas aeruginosa* B18—A plant growth-promoting bacterium with antagonistic potential against *Sporisorium scitamineum*. Front. Microbiol. 12, 628376. 10.3389/fmicb.2021.628376 33613496 PMC7894208

[B90] Singh R. K. Singh P. Li H.-B. Guo D.-J. Song Q.-Q. Yang L.-T.. (2020). Plant-PGPR interaction study of plant growth-promoting diazotrophs *Kosakonia radicincitans* BA1 and *Stenotrophomonas maltophilia* COA2 to enhance growth and stress-related gene expression in *Saccharum* spp. J. Plant. Interact. 15, 427–445. 10.1080/17429145.2020.1857857

[B91] Singh V. K. Singh A. K. Kumar A. (2017). Disease management of tomato through PGPB: current trends and future perspective. 3 Biotech. 7, 1–10. 10.1007/s13205-017-0896-1 28730550 PMC5519495

[B92] Smith A. D. Jameson G. N. Dos Santos P. C. Agar J. N. Naik S. Krebs C.. (2005). NifS-mediated assembly of [4Fe−4S] clusters in the N-and C-terminal domains of the NifU scaffold protein. Biochem. 44, 12955–12969. 10.1021/bi051257i 16185064

[B93] Taylor M. B. Goodwin C. S. Karim Q. (1988). Two urease activities with different pH optima in Campylobacter pylori and similar organisms. Fems. Microbiol. Lett. 55, 259–261. 10.1111/j.1574-6968.1988.tb02811.x

[B94] Teschler J. K. Zamorano-Sánchez D. Utada A. S. Warner C. Wong G. Linington R. G.. (2015). Living in the matrix: assembly and control of Vibrio cholerae biofilms. Nat. Rev. Microbiol. 13, 255–268. 10.1038/nrmicro3433 25895940 PMC4437738

[B95] Ullah N. Ditta A. Imtiaz M. Li X. Rizwan M. (2021). Appraisal for organic amendments and plant growth-promoting rhizobacteria to enhance crop productivity under drought stress: A review. J. Agron. Crop Sci. 207, 783–802. 10.1111/jac.12502

[B96] Verma K. K. Song X. P. Zeng Y. Li D. M. Guo D. J. Rajput V. D.. (2020). Characteristics of leaf stomata and their relationship with photosynthesis in *Saccharum officinarum* under drought and silicon application. ACS Omega. 5, 24145–24153. 10.1021/acsomega.0c03820 32984737 PMC7513552

[B97] Vitosh M. Warncke D. Lucas R. J. W. A. S. P. (1994). Zinc determine of crop and soil, Michigan State University Extension. Water Air Soil Pollut. 100, 133–149.

[B98] Wainwright M. (1984). Sulfur oxidation in soils. Adv. Agron. 37, 349–396. 10.1016/S0065-2113(08)60458-7

[B99] Wang D. Xu A. Elmerich C. Ma L. Z. (2017). Biofilm formation enables free-living nitrogen-fixing rhizobacteria to fix nitrogen under aerobic conditions. Isme J. 11, 1602–1613. 10.1038/ismej.2017.30 28338674 PMC5520150

[B100] Wang J. Qu F. Liang J. Yang M. Hu X. (2022). *Bacillus velezensis* SX13 promoted cucumber growth and production by accelerating the absorption of nutrients and increasing plant photosynthetic metabolism. Sci. Hortic. 301, 111151. 10.1016/j.scienta.2022.111151

[B101] Wang Z. Solanki M. K. Yu Z.-X. Anas M. Dong D.-F. Xing Y.-X.. (2021). Genome Characteristics Reveal the Biocontrol Potential of *Actinobacteria* Isolated From Sugarcane Rhizosphere. Front. Microbiol. 12, 797889. 10.3389/fmicb.2021.797889 35003029 PMC8740303

[B102] Wang Z. Solanki M. K. Yu Z.-X. Yang L.-T. An Q.-L. Dong D.-F.. (2019). Draft genome analysis offers insights into the mechanism by which *Streptomyces chartreusis* WZS021 increases drought tolerance in sugarcane. Front. Microbiol. 9, 3262. 10.3389/fmicb.2018.03262 30687260 PMC6338045

[B103] Wayment D. G. Ledet H. J. Torres K. A. White Jr P. M. H. Part B. (2021). Soil dissipation of sugarcane billet seed treatment fungicides and insecticide using QuEChERS and HPLC. J. Environ. Sci. 56, 188–196. 10.1080/03601234.2020.1858685 33499735

[B104] Wei C.-Y. Lin L. Luo L.-J. Xing Y.-X. Hu C.-J. Yang L.-T.. (2014). Endophytic nitrogen-fixing *Klebsiella variicola* strain DX120E promotes sugarcane growth. Biol. Fertil. Soils 50, 657–666. 10.1007/s00374-013-0878-326203334

[B105] Xia Y. Farooq M. A. Javed M. T. Kamran M. A. Mukhtar T. Ali J.. (2020). Multi-stress tolerant PGPR *Bacillus xiamenensis* PM14 activating sugarcane (*Saccharum officinarum* L.) red rot disease resistance. Plant Physiol. Bioch. 151, 640–649. 10.1016/j.plaphy.2020.04.016 32339911

[B106] Xu Y. B. Chen M. Zhang Y. Wang M. Wang Y. Huang Q. B.. (2014). The phosphotransferase system gene ptsI in the endophytic bacterium *Bacillus cereus* is required for biofilm formation, colonization, and biocontrol against wheat sharp eyespot. Fems Microbiol. Lett. 354, 142–152. 10.1111/1574-6968.12438 24750250

