# Supplementary material for: Complete genome analysis of sugarcane root associated endophytic diazotroph Pseudomonas aeruginosa DJ06 revealing versatile molecular mechanism involved in sugarcane development
Source: Front Microbiol. 2023 Apr 20;14:1096754. doi: 10.3389/fmicb.2023.1096754 (PMC10157262; doi:10.3389/fmicb.2023.1096754)
Supplement: Supplementary file 1 [file Data_Sheet_1.docx]

## Supplementary Tables

**TABLE S1** List of media used for isolation of endophytic strains from sugarcane roots.

| **Media** | \| **Composition** \|  \| \| --- \| --- \| | **Unit (**g/L**)** |
| --- | --- | --- | --- | --- |
| Ashby’s Mannitol Agar | Mannitol | 20 |
|  | Dipotassium hydrogen phosphate | 0.2 |
|  | Magnesium sulphate | 0.2 |
|  | Sodium chloride | 0.2 |
|  | Calcium carbonate | 5 |
|  | Potassium sulphate | 0.1 |
|  | Agar | 15 |
|  | Final pH | 7.4 |
| Burk’s Medium | Magnesium sulphate | 0.2 |
|  | Dipotassium hydrogen phosphate | 0.8 |
|  | Potassium dihydrogen phosphate | 0.2 |
|  | Calcium sulphate | 0.13 |
|  | Iron (III) Chloride | 1.45 mg |
|  | Sodium molybdate | 0.253 mg |
|  | sucrose | 20 |
| Jensen’s Agar | Magnesium sulphate | 0.5 |
|  | Dipotassium hydrogen phosphate | 1 |
|  | Sodium Chloride | 0.5 |
|  | Ferrous sulphate | 0.1 |
|  | Sodium molybdate | 0.005 |
|  | Calcium carbonate | 2 |
|  | Sucrose  Agar | 20  15 |
| Pikovskaya’s Agar | Ferrous sulfate | 1 |
|  | Glucose | 10 |
|  | Magnesium sulfate | 0.1 |
|  | Calcium phosphate | 5 |
|  | Ammonium sulfate | 0.5 |
|  | Potassium chloride | 0.2 |
|  | Manganese sulfate  Agar | 1  15 |
| Yeast Mannitol Agar | Yeast extract | 1 |
|  | Mannitol | 10 |
|  | Sodium chloride | 0.1 |
|  | Calcium carbonate | 1 |
|  | Dipotassium hydrogen phosphate | 0.5 |
|  | Magnesium sulphate | 0.2 |
|  | Agar | 15 |
|  | Final pH | 6.8 |

**TABLE S2** The composition of C_2_ medium

| **Composition** | **Unit (g/L)** |
| --- | --- |
| Peptone | 10 |
| Yeast extract | 5 |
| Sodium chloride (NaCl) | 5 |
| Glucose | 15 |
| Final pH | 6 |





**FIGURE S1** Isolation of endophytic bacteria strains from sugarcane roots using five media

##
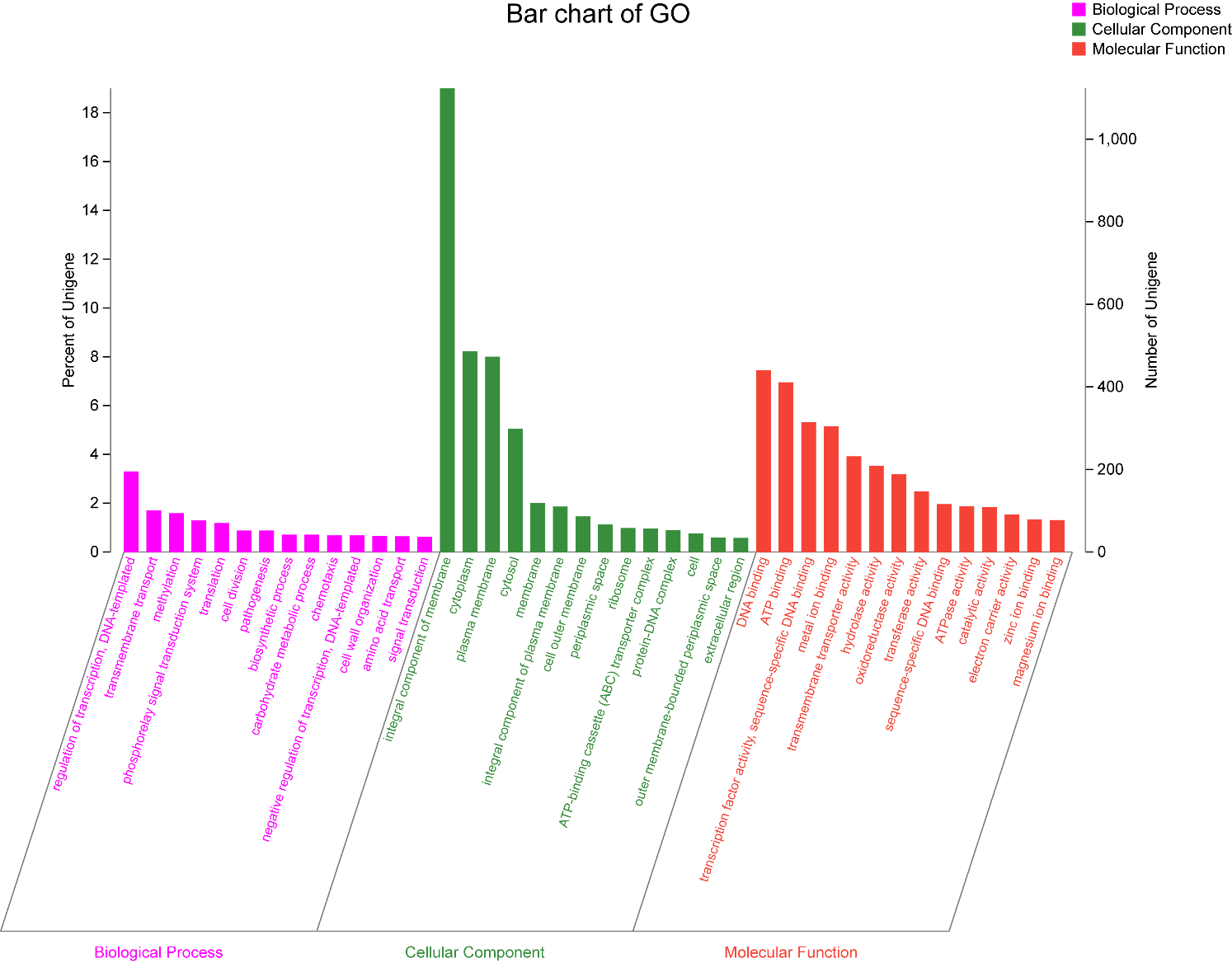
Supplementary Figures

**FIGURE S2** The GO annotations of CDS genes of *Pseudomonas aeruginosa* DJ06 genome.

**
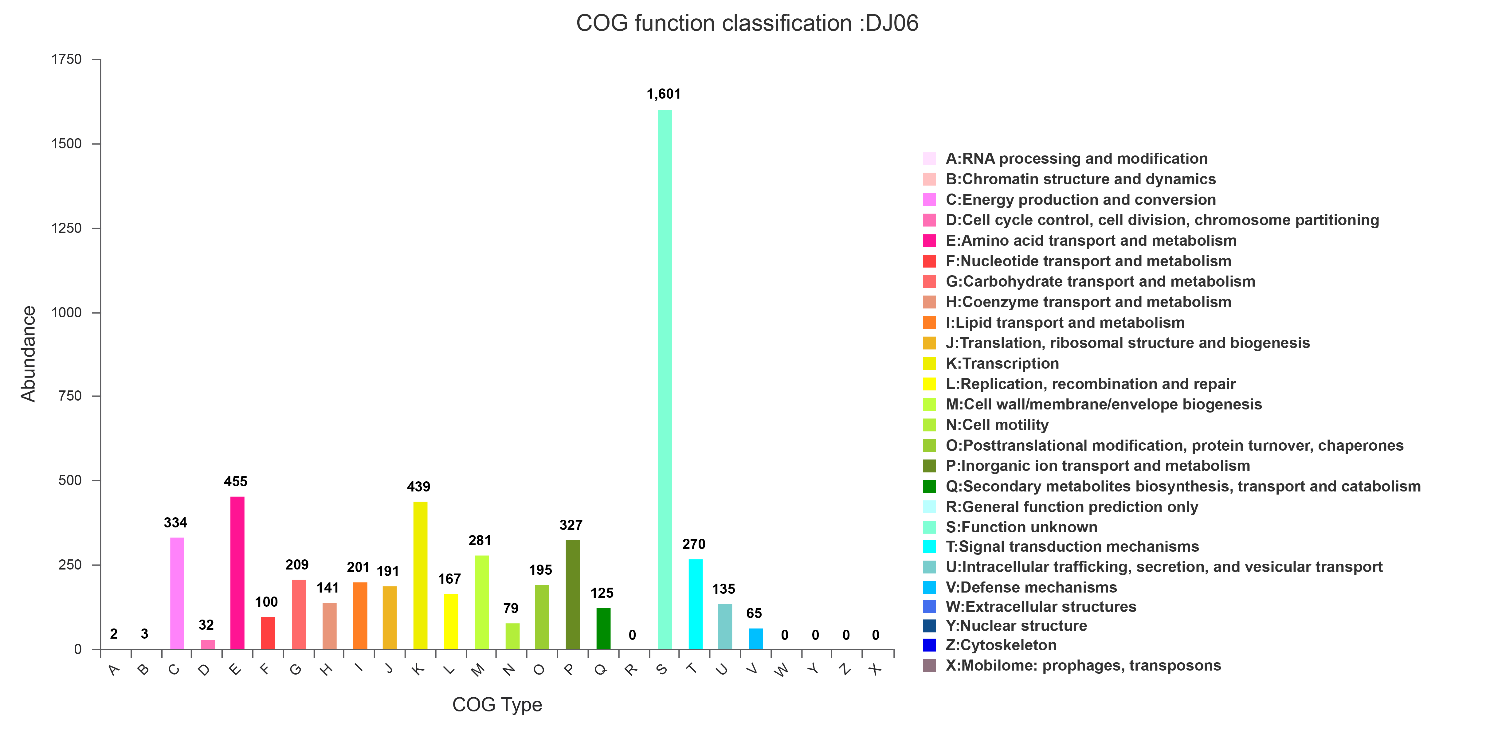
FIGURE S3**The COG annotations of CDS genes of *Pseudomonas aeruginosa* DJ06 genome.

**

FIGURE S4** The KEGG pathway annotations of CDS genes of *Pseudomonas aeruginosa* DJ06 genome.





**FIGURE S5** The plant growth status of sugarcane varieties GT11 (A) and B8 (B) at 60 days after inoculation with strain DJ06
